# Structure and substrate recognition by the bacterial twin-arginine translocation (Tat) core complex

**DOI:** 10.1038/s41564-026-02399-z

**Published:** 2026-06-22

**Authors:** Justin C. Deme, Owain J. Bryant, Mariana R. B. Batista, Phillip J. Stansfeld, Ben C. Berks, Susan M. Lea

**Affiliations:** 1 Center for Structural Biology, Center for Cancer Research, National Cancer Institute, Frederick, MD USA; 2 Sir William Dunn School of Pathology, University of Oxford, Oxford, UK; 3 Central Oxford Structural Molecular Imaging Centre, University of Oxford, Oxford, UK; 4 Department of Biochemistry, University of Oxford, Oxford, UK; 5 Structural Biology, St Jude Children’s Research Hospital, Memphis, TN USA; 6 School of Life Sciences and Department of Chemistry, University of Warwick, Coventry, UK

**Keywords:** Cryoelectron microscopy, Bacterial secretion

## Abstract

The twin-arginine translocation (Tat) system is a mechanistically unique protein transport pathway moving folded proteins across membranes. It is found in all domains of life and is essential for bacterial virulence and plant photosynthesis. The membrane proteins, TatA, TatB and TatC form a core complex to which substrate proteins bind, triggering the recruitment of additional TatA protomers to form the transport site. Here we present cryo-electron microscopy structures of the prototypical TatBC complex from *Escherichia coli* and the atypical complexes from *Nitratifactor salsuginis* and *Myxococcus xanthus* in a resting state, alongside TatAC substrate-bound TatBC and TatABC complexes from *E. coli* in the early stages of transport. These structures demonstrate that substrate proteins associate with the core complex solely through their N-terminal signal peptides. The Tat targeting sequences of the signal peptides make specific contacts with TatC, and the peptide body is clamped by TatB. The core complex contains highly tilted transmembrane helices that drive extreme local membrane thinning. On the basis of our structures and biochemical and functional analyses, we propose a model for the early steps in Tat transport.

## Abstract (web summary)

Cryo-EM structural, biochemical and functional analyses of the bacterial twin-arginine translocation system for protein transport across membranes reveal mechanisms of substrate binding.

## Main

**Fig. 1:**
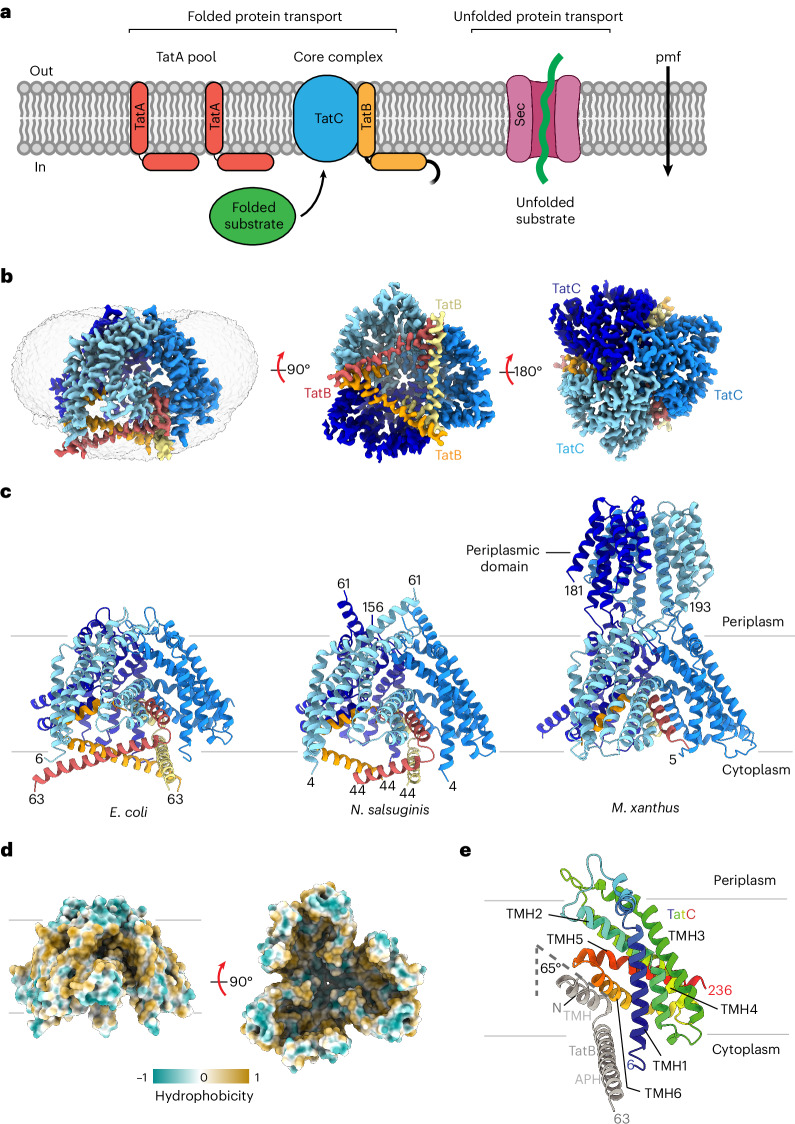
Architecture of the TatB_3_C_3_ complex. **a**, The Tat system transports folded substrates across membranes and consists of a core complex minimally composed of several copies of TatB and TatC, and a large pool of TatA monomers. Substrate docking to the core complex promotes the pmf-dependent recruitment of multiple TatA protomers to form the active translocation site. Right: by contrast, the Sec system found in the same membrane transports proteins in the unfolded state. **b**, The cryo-EM volume of the *E. coli* TatBC complex in GDN at 3.1 Å resolution (contour level 0.175), viewed from the membrane plane (left), from the cytoplasm (middle) and periplasm (right). Left: the detergent micelle (grey, contour level 0.08) indicates the position of the membrane. The APHs of the three copies of TatB form the triangle of helices visible on the base of the complex in the centre panel. **c**, Cartoon models of TatBC complexes from *E. coli*, *N. salsuginis* and *M. xanthus* viewed from the plane of the membrane. The large periplasmic insert in *N. salsuginis* TatC is disordered and not resolved. A membrane, assigned from the position of the detergent micelle, is depicted by thin lines in this and in **d** and **e**. **d**, The surface model of the TatC trimer within the *E. coli* TatBC complex coloured by amino acid hydrophobicity. **e**, A cartoon representation of a single TatBC heterodimer from within the *E. coli* TatBC complex. TatC is coloured from blue at the N-terminus to red at the C-terminus, and TatB is coloured grey. The angle the TatB TMH makes with the membrane normal is shown.

Bacteria use parallel Sec and twin-arginine translocation (Tat) protein transport pathways to export proteins across their cytoplasmic membrane. These pathways are conserved in the thylakoid membrane of plant chloroplasts, in certain mitochondria and, in the case of Sec, also the eukaryotic endoplasmic reticulum, making them the most widely distributed protein transport systems in biology^1–9^. Although Sec transport is well understood, the Tat pathway^2,10–12^ remains poorly characterized despite its critical roles in the biogenesis of respiratory and photosynthetic machineries, cell envelope formation and pathogenesis^1–3,13^. Tat substrates are characterized by N-terminal signal peptides containing a distinctive twin-arginine motif^14–16^ that interacts with TatC^17–24^. Uniquely among protein transport systems that reside in ion-impermeable membranes, the Tat pathway transports its substrate proteins in a folded rather than unfolded state^3,14,25^ (Fig. 1a), posing the challenge of moving substrates of varying sizes and shapes without compromising membrane integrity.

In most organisms, the minimal components of the Tat system are the three integral membrane proteins TatA, TatB and TatC^26–28^ (Fig. 1a). TatA and TatB are structurally related but functionally non-identical proteins that both contain a single N-terminal transmembrane helix (TMH) followed by an amphipathic helix (APH)^29–31^. TatC contains six TMHs^19,32^ and, together with TatB, forms a multimeric core complex that engages with the signal peptides of substrate proteins^20,21,33,34^. TatA protomers may also be part of this core complex^34,35^ but have been thought to be peripherally associated in the resting state^36,37^.

The docking of a substrate protein to the core complex triggers a protonmotive force (pmf)-dependent recruitment and oligomerization of many additional TatA subunits to form the translocation site through which the folded substrate is transported^17,38–40^. Tat transport is not thought to utilize a conventional protein-conducting channel, but instead, the TatA oligomer is proposed to perturb the membrane bilayer structure to allow the transmembrane movement of the substrate protein^29,41–44^. The completion of transport is followed by the disassembly of the translocation site and the removal of the signal peptide from the substrate protein by signal peptidase.

The architecture of the Tat core complex and how it recognizes substrates remain unknown. Here, we use cryo-electron microscopy (cryo-EM) to determine structures of the core complex alone and bound to substrate proteins.

## Results

### The TatBC complex

**Extended Data Fig. 1:**
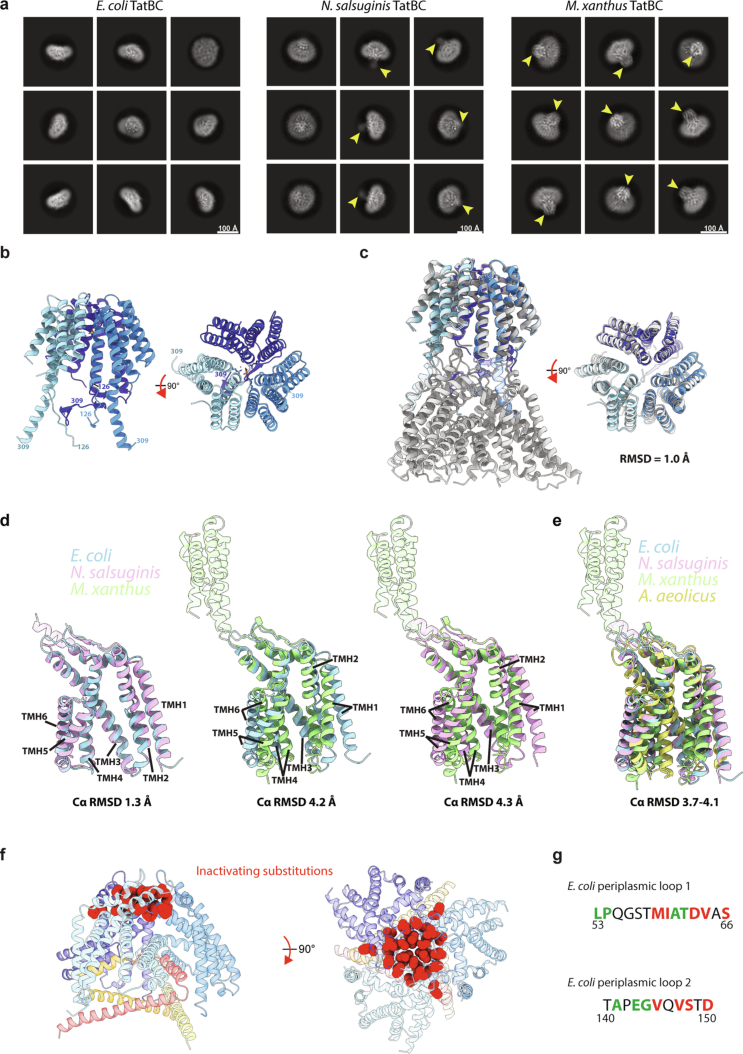
Architectures of TatBC complexes. **a**, Cryo-EM 2D class averages of TatBC complexes purified in GDN from *E. coli* (left), *N. salsuginis* (middle), and *M. xanthus* (right). The yellow arrowheads indicate the location of the periplasmic domains in the *N. salsuginis* and *M. xanthus* complexes. **b**, Cartoon representation of the structure of the isolated periplasmic domain of *M. xanthus* determined by X-ray crystallography with each subunit coloured in different shades of blue. A sulphate atom bound on the three^−^fold axis together with coordinating side chains and waters are shown in stick representation coloured by chemical composition **c**, Structural alignment of the *M. xanthus* periplasmic domain solved by X-ray crystallography (coloured as in **b**) and the TatBC complex solved by cryo-EM (grey). Residues 135-289 were used for alignment. Those parts of the crystal structure not used in the alignment are shown transparent. **d**, Pairwise structural alignments of the TatC models presented in this study. **e**, Structural alignment of the TatC models presented in this study against the crystal structure of *A. aeolicus* TatC (yellow; PDB 4B4A). Periplasmic residues that did not contribute to the alignment are shown with increased transparency. **f**, Model of the *E. coli* TatBC complex viewed from the plane of the membrane (left) or the periplasm (right). Residues within the periplasmic loops of TatC where mutations have previously been shown to be important for core complex function or assembly^21,47^ are shown in red space filling representation. **g**, Positions within the TatC periplasmic loops coloured according to whether previously reportedsubstitutions^47,51,52^ inactivate (red) or have no effect (green) on Tat transport. Positions not tested are coloured black.

To obtain the structure of the Tat core complex using single-particle cryo-EM, we initially focused on core complexes containing just TatB and TatC to avoid possible heterogeneity associated with the presence of TatA. We initially targeted the prototypical TatBC complex from *Escherichia coli*. However, the structural analysis of this complex proved challenging owing to its small size and minimal features outside the solubilizing detergent micelle. We thus also targeted atypical Tat complexes from *Nitratifractor salsuginis* and *Myxococcus xanthus* that contain large inserts in different TatC periplasmic loops that might aid structure determination. Additional protein density outside the detergent micelle was indeed visible in two-dimensional (2D) classes from single-particle cryo-EM data of the TatBC complexes of both species (Extended Data Fig. 1a), and the *M. xanthus* insert folded to form a homotrimer for which we determined a structure by X-ray crystallography (Extended Data Fig. 1b).

**Extended Data Fig. 2:**
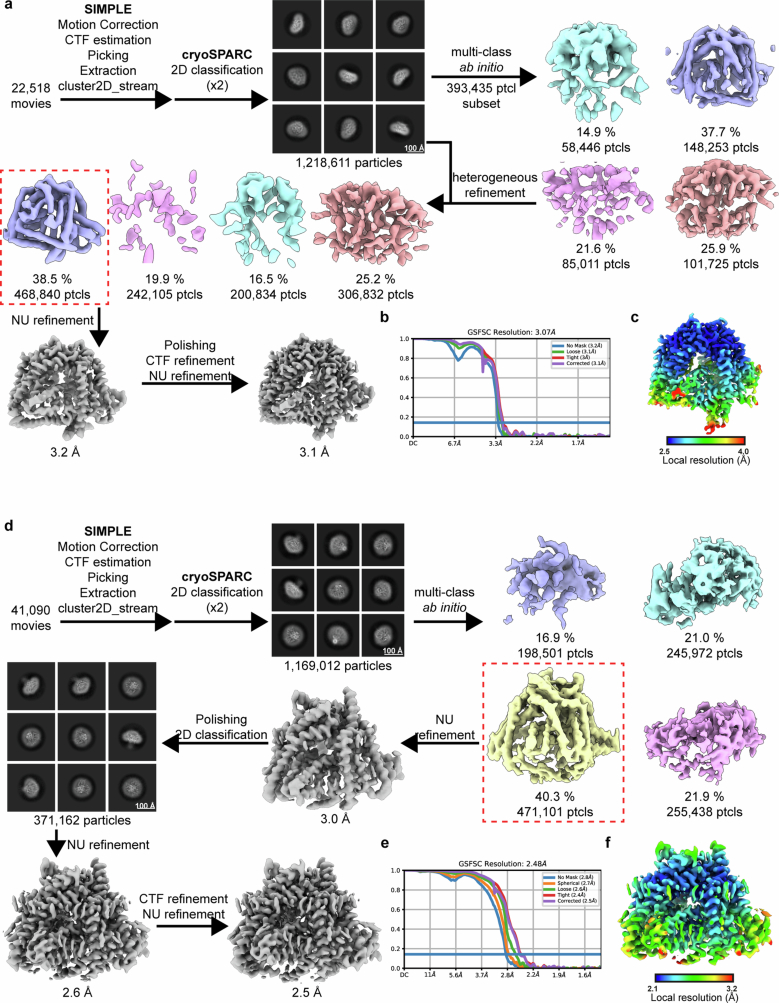
Cryo-EM workflows for *E. coli* and *N. salsuginis* TatBC complexes. *E. coli* **(a-c)** and *N. salsuginis* **(d-f)** cryoEM workflows. **a,d**, Image processing workflow. **b,e**, Gold-standard Fourier Shell Correlation (FSC) curves used for global resolution estimation. **c,f**, Local resolution estimate of the volume.

**Extended Data Fig. 3:**
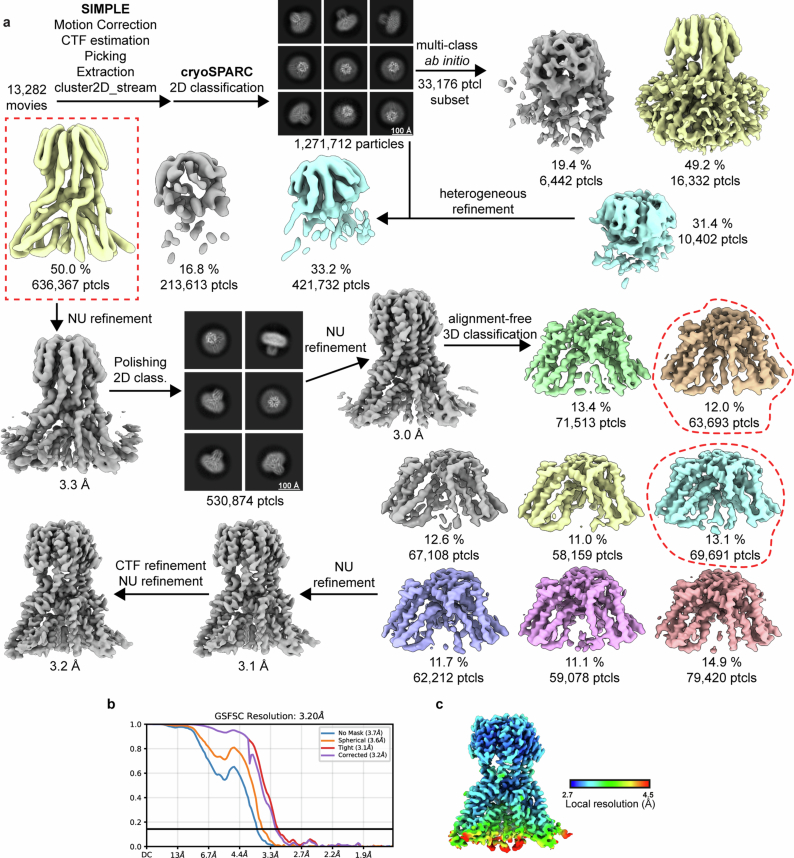
Cryo-EM workflow for the *M. xanthus* TatBC complex. **a**, Image processing workflow. **b**, Gold-standard Fourier Shell Correlation (FSC) curves used for global resolution estimation. **c**, Local resolution estimate of the volume.

We resolved structures of all three TatBC complexes at 2.6–3.1 Å (Fig. 1b,c and Extended Data Figs. 1–3) revealing a common (TatBC)_3_ architecture in which the complex is formed from a trimer of TatBC heterodimers and clarifying a stoichiometry that had previously been overestimated^11,20,33,35,45,46^. The periplasmic insert in TatC in the *M. xanthus* complex is ordered and folded as in the crystal structure (Fig. 1c and Extended Data Fig. 1c), but the unrelated TatC insert in the *N. salsuginis* complex is disordered (Fig. 1c and Extended Data Fig. 1a). The remaining parts of TatC could be built in their entirety except for a small number of residues at the polypeptide termini and in several interhelix loops. In the better-ordered *E. coli* and *N. salsuginis* complexes, the TMH and APH of TatB were resolved (in the *M. xanthus* complex only the TM could be built), suggesting that residues C-terminal to the APH are disordered or mobile relative to the membrane-anchored portion of the complexes. Unless otherwise indicated, we henceforth specifically reference the *E. coli* TatBC complex, in which TatB residues 1–63 and TatC residues 6–236 can be modelled, but the three complexes are highly similar (Extended Data Fig. 1d)

The three copies of TatC within the TatBC complex each have a ‘glove’ shape and are arranged with their concave faces directed to the centre of the complex and their convex faces facing out towards the lipid bilayer. Compared with previous *A. aeolicus* TatC monomeric crystal structures^19,32^, the TatC proteins in the TatBC complex exhibit a subtle closure of the C-terminal end of the TatC ‘glove’, leading to an increase in concavity (Extended Data Fig. 1e). The three TatC molecules pack tightly at the periplasmic side of the membrane, forming an inverted cup-like structure in which the largely hydrophobic interior surface is open to the cytoplasm (Fig. 1d). The observed TatC–TatC contacts include residues previously identified as important for core complex function or assembly^21,47^ (Extended Data Fig. 1f,g). In the *M. xanthus* TatBC complex, the TatC interactions are augmented by the contacts between the inserted periplasmic domains (Fig. 1c and Extended Data Fig. 1c).

**Fig. 2:**
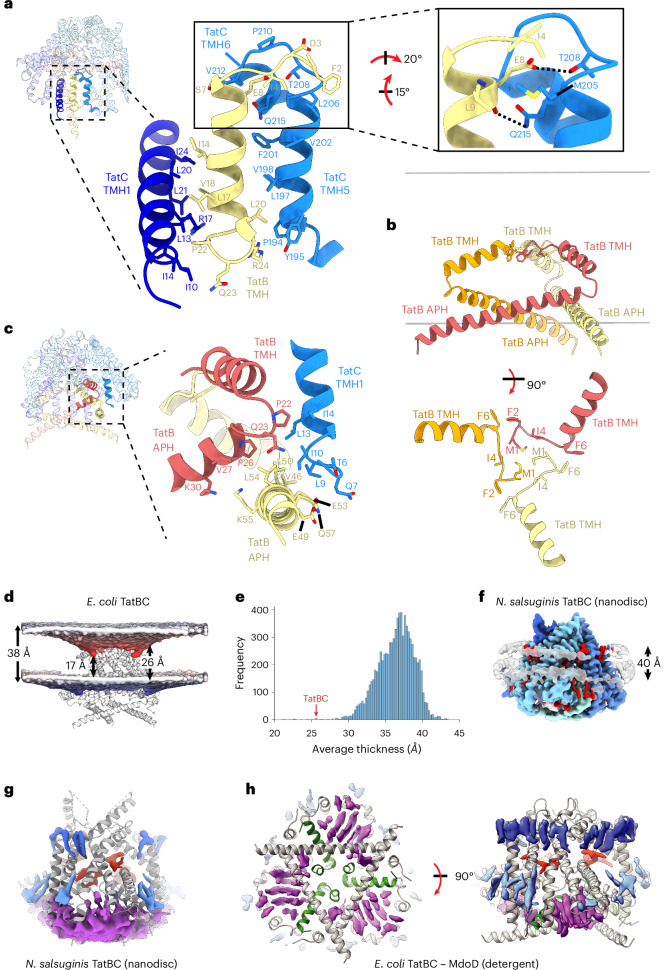
TatBC complex assembly and membrane thinning. **a**, The structure of an *E. coli* TatC-TatB-TatC interface. Shown are TMH1 of the first TatC monomer (dark blue), TMH5 of the second TatC monomer (light blue) and the TatB TMH (yellow). Right, inset: the zoomed-in view highlights the interactions found at the previously proposed ‘polar cluster site’. Hydrogen bonds are depicted as dashed lines. **b**, The structure of the TatB cage formed by the three copies of TatB (coloured in red, orange and yellow). Top: the view from the membrane plane. Bottom: the view of the three TatB TMHs viewed from the cytoplasm. **c**, The structure of an *E. coli* TatB–TatB–TatC interface. TatC TMH1 is shown in light blue; two TatB copies are shown in red and yellow. **d**, Atomistic MD simulation of the *E. coli* TatBC complex in a membrane bilayer highlighting the membrane thinning induced by the TatBC complex. **e**, The distribution of average membrane thickness values for the annular shell (within 7 Å) around the membrane proteins in the MemProtMD database. The average thickness for TatBC is highlighted. **f**, The TatBC complex thins a membrane-mimetic bilayer. The Cryo-EM volume of the *N. salsuginis* TatBC complex in a lipid nanodisc at a resolution of 3.1 Å, viewed from the membrane plane (contour level 0.222). Lipid densities are coloured red and the helical nanodisc belts grey. **g**,**h**, Lipid or detergent densities visible in the cryo-EM volumes of the *N. salsuginis* TatBC complex (at 2.9 Å resolution) (**g**) or an *E. coli* TatBC–substrate complex (at 2.6-Å resolution where the lipid densities are better defined) (**h**). The densities are coloured according to their position in the complex: either vertically oriented at the base of the TatC cup (purple), horizontally oriented higher in the TatC cup (red) or on the outside of the complex in either the periplasmic (dark blue) or cytoplasmic (light blue) leaflet of the membrane.

**Extended Data Fig. 4:**
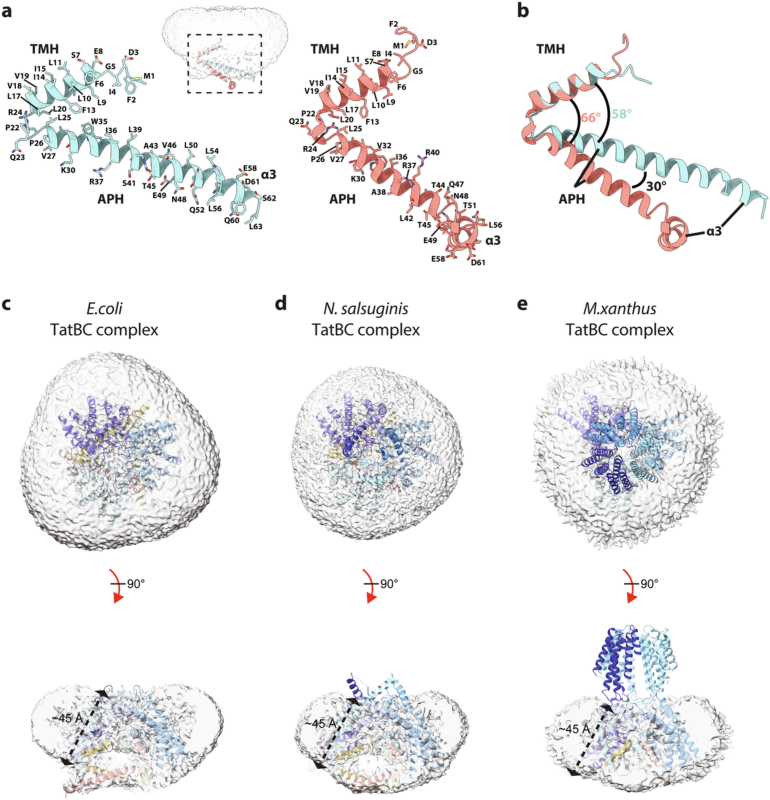
TMH tilting leads to thinning of the detergent micelle around the TatBC complex. **a**,**b**, The arrangement of the TatB helices observed in the TatBC complex (sky blue) differs from that in an earlier NMR structure of isolated TatB (salmon, PDB 2MI2). The structures are shown separately (**a**) or superimposed (**b**, aligned on TMH residues 6-21, Cα RMSD ~ 0.5 Å). The inset in (**a**) shows the NMR structure superimposed on the position of TatB in the TatBC complex. Alignments and RMSD were calculated in ChimeraX^85^. **c-e**, Tilting of the TatC TMHs thins the hydrophobic layer around the TatBC complex as seen in the detergent/phospholipid micelles around the purified complexes. Models of the *E. coli* (**c**), *N. salsuginis* (**d**) and *M. xanthus* (**e**) TatBC complexes superposed with low contoured cryo-EM volumes to display the detergent/phospholipid micelle (grey). TatB density is depicted in red, orange and yellow, TatC density in various shades of blue.

The TMH and APH of TatB in the TatBC complex span the same residues as in an earlier NMR structure of the isolated protein^30^, although with the small α3 helix identified by NMR now extending the APH and with the orientation of the two helices rearranged relative to the NMR structure (Extended Data Fig. 4a,b). Each TatB subunit is intercalated between two TatC subunits (Figs. 1b,e and 2a) consistent with earlier biochemical predictions^19,21,34^. This arrangement is stabilized primarily by interactions between the TatB TMH and two regions of neighbouring TatC molecules: TMH5 and the TMH5-6 loop of one adjacent TatC and TMH1 of the other (Fig. 2a and Supplementary Fig. 1a).

The three TatB APHs form a triangular arrangement spanning the open cytoplasmic face of the TatC cup (Fig. 1b), with each APH packing against its neighbour and the N-terminus of TatC TMH1 (Fig. 2b,c). Together, the ordered TatB regions form a cage-like structure (Fig. 2b) contained within the TatC trimer (Fig. 1c).

The N-terminal end of the TatB TMH contains the highly conserved polar residue E8. This amino acid was previously inferred to stabilize the association of TatB with TatC through interaction with a ‘polar cluster site’ on TatC formed by M205, T208 and Q215^34^. Our TatBC structure reveals that although the side chain of TatC T208 forms the anticipated hydrogen bond with the side chain of TatB E8, the side chain of Q215 hydrogen bonds to the main chain oxygen of E8 rather than making the predicted interaction with the side chain of this residue (Fig. 2a). TatC M205 does not directly contact TatB E8 but packs between TatB L9 and I4. The TatC molecule that provides the polar cluster interactions makes many further contacts to the E8-containing face of the TatB TMH through a track of hydrophobic residues along the face of TatC TMH5, the TMH5-6 loop and the N-terminal end of TMH6 (Fig. 2a). The other side of the TatB TMH is stabilized by a series of hydrophobic interactions with TMH1 of the other neighbouring copy of TatC (Fig. 2a,c). These include previously identified contacts between TatC L21 and TatB V18^34^ (Fig. 2a and Supplementary Fig. 1a) and interactions with TatB made by TatC I10 and I14^21^ (Fig. 2a,c and Supplementary Fig. 1a).

The TMHs of both TatB and TatC are highly tilted relative to the membrane normal (Figs. 1e and 2b) rather than having the near-vertical arrangement suggested by simulations of the TatC monomer in lipid bilayers^19,32^. In the case of the TatB TMHs, the N-termini of the TatB molecules pack together within the TatC cup at the midplane of the transmembrane portion of the complex (Fig. 2b) with the TMHs projecting outwards towards the cytoplasmic end of the TatC–TatC interfaces at an angle of 65° relative to the membrane normal (Fig. 1e). This extreme TMH tilting is matched by TMH5 and 6 of TatC, which also penetrate only halfway across the membrane-spanning part of the complex from the cytoplasm (Fig. 1e).

The extensive TMH tilting compresses TatBC along the membrane normal (Fig. 1c and Extended Data Fig. 4c–e), which molecular dynamics (MD) simulations show causes dramatic local membrane thinning from 38 Å in the bulk bilayer to just 26 Å adjacent to the complex (Fig. 2d). This level of membrane thinning is extreme relative to all other helical membrane proteins in the MemProtMD database (Fig. 2e).

**Extended Data Fig. 5:**
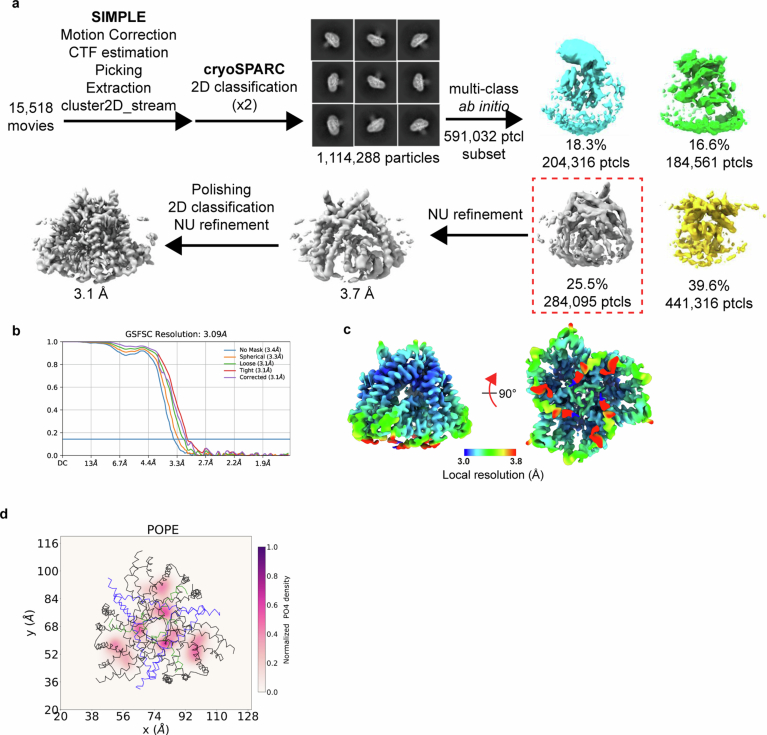
Cryo-EM workflow for the *N. salsuginis* TatBC complex in a nanodisc and the distribution of lipids in an MD simulation of *E. coli* TatBC. **a-c**, CryoEM workflow for the *N. salsuginis* TatBC complex in a nanodisc. **a**, Image processing workflow. **b**, Local resolution estimate of the volume. **c**, Gold-standard Fourier Shell Correlation (FSC) curves used for global resolution estimation. **d**, coarse^−^grained MD simulation of *E. coli* TatBC in a model membrane bilayer. Density plot of the lipids located within the TatBC complex, initial positions were assigned by docking into low resolution experimental density. The lipids remain stably associated within the complex and do not diffuse into the bulk membrane. Data are derived from three independent simulations of 5 µs each (n = 3000 MD frames).

MD simulations indicate that the compressed TatBC structure is stable in a membrane environment and not an artefact of detergent solubilization (Supplementary Fig. 2). This was confirmed experimentally by showing the *N. salsuginis* complex is structurally identical in nanodiscs and detergent micelles (root mean square deviation 0.8 Å for all CA pairs; Fig. 2f and Extended Data Fig. 5a–c). The bilayer around TatBC in the nanodiscs is ~40 Å wide, compared with ~60 Å for the native *E. coli* membrane^48^, consistent with local bilayer thinning by the complex.

Both nanodisc (Fig. 2g) and detergent-solubilized (Fig. 2h) structures reveal extensive ordered lipid densities around and within TatBC. Several lipids stack against the tilted TMHs (Fig. 2g,h, blue densities), others occupy cavities within the TatC concave faces (Fig. 2g,h, purple densities) and a pair of lipids per TatC subunit are oriented horizontally at the periplasmic end of the TatC cup (Fig. 2g,h, red densities). MD simulations observe lipid accumulation at these same sites (Extended Data Fig. 5d). The interior lipids adopt an arrangement markedly different from the surrounding bilayer. The high abundance and order of the interior lipids, together with the sensitivity of the complex to all but the most gentle detergents^33,49^, suggests that lipids play a key role in stabilizing the assembly.

### Structural basis for signal peptide recognition by the TatBC complex

**Fig. 3:**
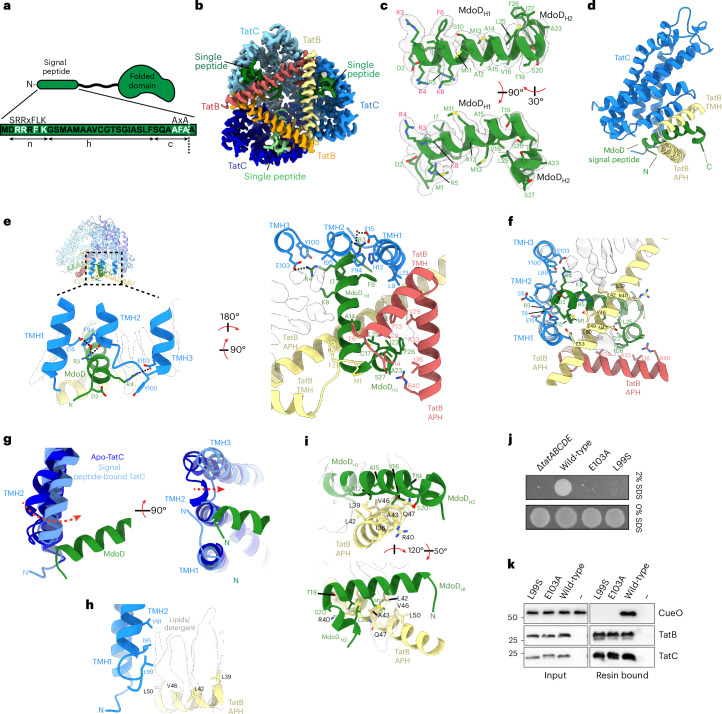
Signal peptide recognition by the TatBC complex. **a**, The features of a Tat signal peptide shown schematically (top) or identified within the signal peptide of MdoD (bottom). The site of cleavage by signal peptidase is indicated by a dashed line. **b**, The Cryo-EM volume of the *E. coli* TatBC–MdoD complex viewed from the cytoplasm. Signal peptide (green, contour level 0.17). **c**, A model for the MdoD signal peptide shown in the cryo-EM volume (transparent grey surface). **d**, One TatBC–signal peptide unit viewed from the membrane plane. **e**, The views from a position in the membrane plane (left) and from within the interior of the complex facing towards the cytoplasm (right). **f**, A view from the cytoplasm. **g**, The structural alignment of the apo- and signal peptide-bound TatBC complexes (dashed red arrow shows movements on signal peptide binding). The selected portion of the complex is viewed from the membrane plane (left) or the cytoplasm (right). **h**, TatC Leu-99 is close to lipid or detergent densities within the TatC lipid cavity. **i**, Residues forming the signal peptide–TatB APH interface. In **d**–**i**, various views of details of the TatBC–MdoD complex with proteins are shown as cartoon representations and lipid/detergent densities in white. **j**,**k**, TatC L99S is required for Tat activity. *E. coli* strains expressing the chromosomal copy of *tatC* with a C-terminal Twin-Strep affinity tag and the indicated single amino acid substitutions were analysed. **j**, Tat function was assessed through the ability to grow in the presence of SDS. Strains expressing TatC E103A^52^ or deleted for all *tat* genes (Δ*tatABCDE*) were used as transport-inactive controls. **k**, The ability of the variant TatBC complexes to bind substrate molecules was assessed by asking whether the Tat substrate protein CueO is pulled from solution by resin-immobilized TatBC complexes. The proteins were detected by immunoblotting. A TatC(E103A) variant was used as a binding-negative control^40^. Source data

Tat signal peptides are composed of a positively charged n-region that includes the pair of adjacent arginine residues, a hydrophobic h-region and a polar c-region that contains a cleavage site for signal peptidase^14^ (Fig. 3a). In bacterial Tat signal peptides, the pair of adjacent arginine residues are part of a larger SRRxFLK consensus motif^14,25^.

**Extended Data Fig. 6:**
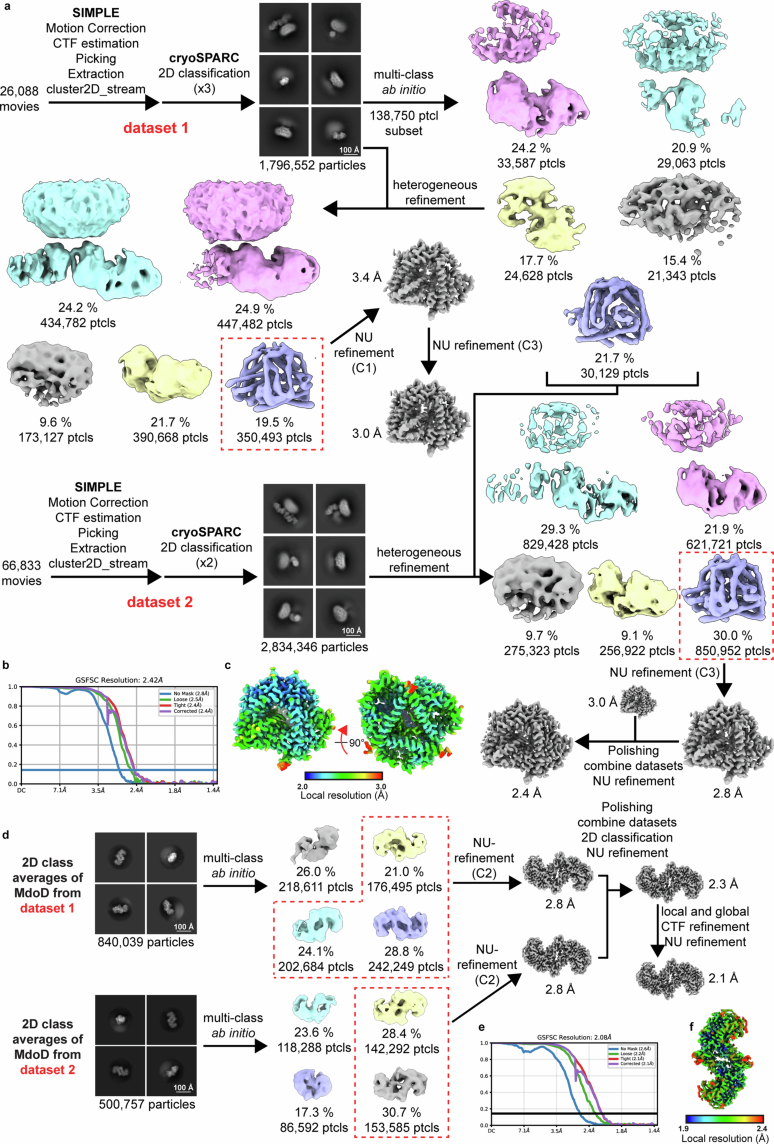
Cryo-EM workflow for the *E.coli* TatBC–MdoD complex and MdoD dimer. *E. coli* TatBC–MdoD complex **(a-c)** and MdoD dimer **(d-f)** cryoEM workflows. **a,d**, Image processing workflow. **b,e**, Gold-standard Fourier Shell Correlation (FSC) curves used for global resolution estimation. **c,f**, Local resolution estimate of the volume.

**Extended Data Fig. 7:**
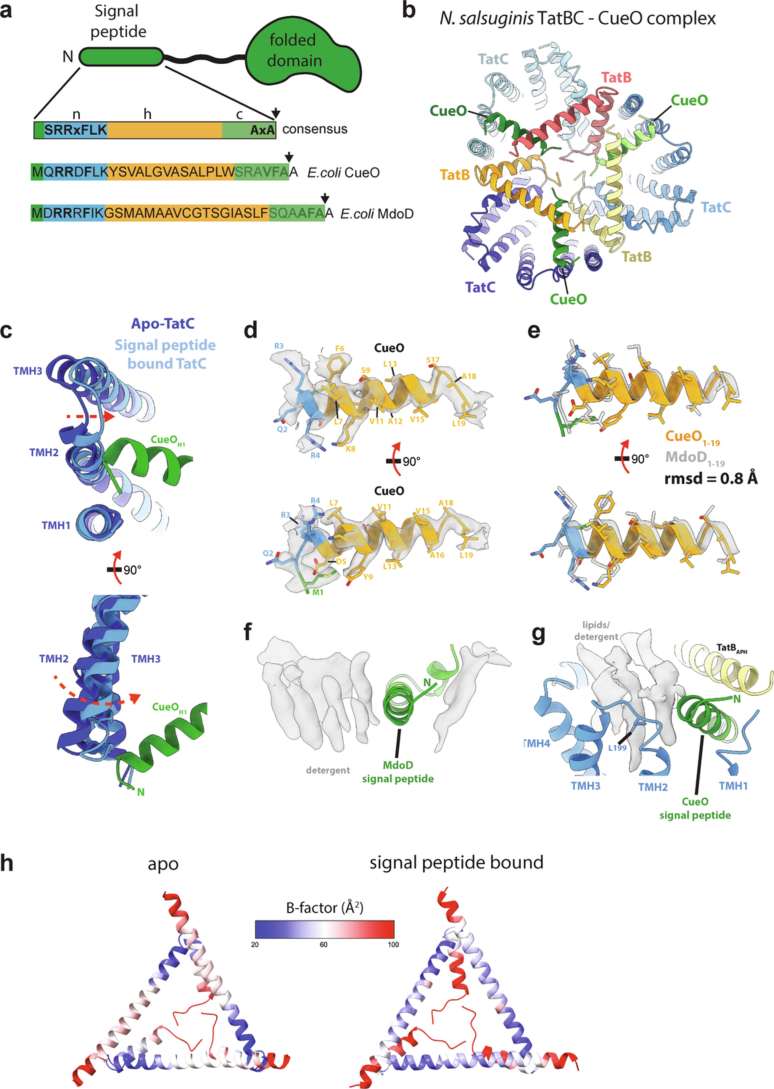
Signal peptide recognition by the TatBC complex. **a**, The features of a Tat signal peptide shown schematically (top) and identified within the sequences of the signal peptides of the *E. coli* proteins CueO and MdoD (below). The site of cleavage by signal peptidase is indicated by a black arrow. **b-f**, Structural analysis of the *N. salsuginis* TatBC-CueO signal peptide complex. **b**, Model of the complex viewed from the cytoplasm. **c**, Structural alignment of the signal peptide^−^bound (TatC light blue and signal peptide green) and apo (TatC dark blue) TatBC complexes (arrow shows movements on signal peptide binding). The selected portion of the complex is viewed from the cytoplasm (top) or the membrane plane (bottom). **d**, Model for the CueO signal peptide shown in the cryo-EM volume (transparent grey surface) with the peptide coloured as in (a). **e**, Structural alignment of the CueO signal peptide from the *N. salsuginis* TatBC-CueO signal peptide complex against the MdoD signal peptide from the *E. coli* TatBC-MdoD complex. Alignments and RMSD were calculated using the MatchMaker command in ChimeraX^85^. **f**,**g**, Lipids are packed between TatC and the signal peptide. *N. salsuginis*Leu199 (in sticks representation) is the equivalent of *E. coli* Leu99 (Fig. 3h). **h**, The ordering of the TatB APH increases on signal peptide binding. Cartoon representation of TatB coloured by B factors for the apo (left) and MdoD-bound (right) *E. coli* TatBC complexes.

To understand how the TatBC complex recognizes signal peptides, we collected cryo-EM data from complexes assembled in vitro between TatBC and either substrate proteins or isolated signal peptides. The highest-resolution structure (2.4 Å) was of *E. coli* TatBC bound to three copies of the substrate MdoD (Fig. 3b and Extended Data Fig. 6a–c). All three signal peptides were clearly resolved at the cytoplasmic face of the complex, enabling confident model building (Fig. 3b,c). A lower-resolution structure of *N. salsuginis* TatBC in complex with the *E. coli* CueO signal peptide was also obtained and was consistent with the higher-resolution structure (Extended Data Fig. 7a–f and Supplementary Fig. 3). In both cases, the stoichiometry of the in vitro complexes assembled with excess substrate and in the absence of TatA, was 1:1:1 (TatB:TatC:signal peptide).

The TatBC–MdoD complex structure shows that the bound signal peptide is predominantly helical, with a long helix (H1, residues 2–20) encompassing the n- and h-regions and a shorter helix (H2, residues 22–26) corresponding to the c-region (Fig. 3c). When viewed from the cytoplasm, H1 runs from the periphery of the complex towards the centre (Fig. 3b,d–f), and the n-region interacts with the cytoplasmic ends of TMHs 1, 2 and 3 from a single TatC protomer (Fig. 3e and Supplementary Fig. 1b). The APHs from two neighbouring TatB protomers also interact extensively with the signal peptide (Fig. 3e,f and Supplementary Fig. 1b). The h-region threads through the TatB cage, passing over (as viewed from the cytoplasm) one of the interacting TatB APHs. The turn into the c-region then packs under the N-termini of the two signal peptide-interacting copies of TatB in the centre of the complex (Fig. 3e). From there, the c-region helix (H2) exits the central cavity towards the cytoplasm, while maintaining interactions with the APH of the second TatB molecule (Fig. 3d–f). The hydrophobic h-region makes extensive interactions with lipids packed on both sides of this part of the signal peptide (Fig. 3e,f,h,i).

We observe two structural changes in the TatBC complex upon signal peptide binding. First, a subtle closing of the ends of TatC TMH2 and TMH3 and their connecting loop around the signal peptide n-region (Fig. 3g) driven by bonding interactions with the signal peptide consensus motif (below). Second, the TatB APH becomes more ordered, suggesting a stabilization of its structure in response to signal engagement (Extended Data Fig. 7g). This change in the mobility of the APH upon signal peptide binding would be consistent with a model in which displacement of the APH allows initial signal peptide access to the core complex whereupon the APH clamps onto the now engaged signal peptide to stabilize the binding interaction.

Side chain density for key residues within the signal peptide motif (SRRxFIK) permitted confident modelling (Fig. 3c), allowing us to determine the role of these conserved residues in substrate recognition. The first position in the motif is normally occupied by a residue with high helix-capping propensity^14^ and our structure confirms that this residue (MdoD D2) caps helix H1 (Fig. 3e,f). The side chain of the first arginine in the motif (MdoD R3) is hydrogen-bonded via an intermediary water molecule to E15 in TatC TMH1 and stacks under F94 from TatC TMH2 (Fig. 3e). The second arginine in the motif (MdoD R4) forms a salt bridge to E103 in TatC TMH3 and packs against Y100 in the TMH2-3 loop (Fig. 3e,f). The consensus phenylalanine (MdoD F6) packs into a hydrophobic pocket formed by F94 in TatC TMH2 and L9, H12 and L13 in TatC TMH1 (Fig. 3e). The residues at the consensus leucine and lysine positions (MdoD I7 and K8) interact primarily with lipid densities, with I7 interacting with the hydrophobic tails and K8 positioned to interact with the less well-ordered head groups (Fig. 3e,f). Notably, R4 is also positioned to interact with these lipid head groups (Fig. 3e). The key TatC residues involved in signal peptide recognition (H12, E15, F94, Y100, E103; Fig. 3e) are highly conserved and have previously been implicated in substate binding by biochemical and genetic studies^19,22,23,50^.

TatC residue L99, previously linked to signal peptide recognition^19,23,51,52^, is not in direct contact with the signal peptide but instead helps trap lipids between the core complex and the signal peptide (Fig. 3e,f,h,i). Similar lipid densities appear in the *N. salsuginis* structure (Extended Data Fig. 7f), suggesting that these lipids contribute to signal peptide recognition. Supporting this idea, substituting L99 with a polar serine residue to increase side chain polarity abolishes both Tat function (Fig. 3j) and substrate binding (Fig. 3k).

### Association of full-length substrate proteins with the TatBC complex

**Fig. 4:**
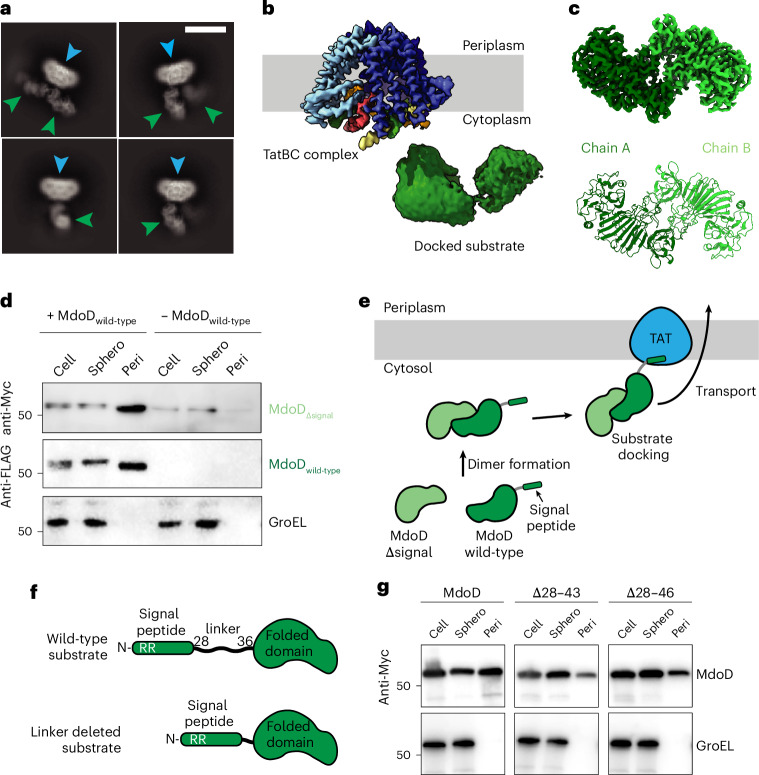
Docking and transport of full-length Tat substrates. **a**, Representative 2D class averages of side views of a TatBC–MdoD complex showing the core TatBC complex (blue arrow) and additional densities corresponding to docked MdoD substrates (green arrow). Scale bar, 100 Å. **b**, The cryo-EM volume of the full TatBC–MdoD complex viewed from the membrane plane. The density corresponding to the TatBC complex and to the bound MdoD substrate is shown at a contour level of 1.2 and 0.55, respectively. The membrane, assigned from the position of the detergent micelle, is depicted by a grey box. **c**, The cryo-EM volume of the *E*. *coli* MdoD dimer at a resolution of 2.1 Å (top) (contour level 0.24) and model (bottom). **d**, A MdoD variant lacking the Tat signal peptide can be exported when coexpressed with signal peptide-bearing MdoD. Transport was assessed in an *E. coli* strain producing a MdoD variant lacking the Tat signal peptide (MdoD_Δsignal_) either with or without the coproduction of full-length MdoD (MdoD_wild-type_). The MdoD proteins were epitope tagged with either a Myc tag (MdoD_Δsignal_) or FLAG tag (MdoD_wild-type_). Whole cells (cell), spheroplast (sphero) and periplasmic (peri) fractions were subjected to immunoblotting with antibodies against the MdoD epitope tags or the cytoplasmic marker protein GroEL. **e**, A schematic showing the process of MdoD_Δsignal_ transport based on the results of **d**. A MdoD dimer formed in the cytoplasm between MdoD_Δsignal_ and MdoD_wild-type_ can engage with the Tat export machinery via the Tat signal peptide on MdoD_wild-type_. **f**, A schematic comparing wild-type MdoD (top) with a linker-deleted MdoD variant (bottom). The N-terminal Tat signal peptide of MdoD is connected to the folded domain by a linker region (residues 28–36). **g**, A comparison of the transport competence of wild-type MdoD and variants with deletions of amino acids 28 to 43 (Δ28–43) or 28 to 46 (Δ28–46). Whole cells (cell), spheroplast (sphero) and periplasmic (peri) fractions were subjected to immunoblotting to identify MdoD or the cytoplasmic marker protein GroEL. Source data

A further analysis of the TatBC–MdoD dataset revealed how substrate folded domains are positioned when their signal peptide is engaged. Reprocessing the data with a larger box produced 2D class averages showing MdoD passenger domains (Fig. 4a, green arrows) located adjacent to the TatBC complex (Fig. 4a, blue arrows). Most classes showed only one or two well-ordered passenger domains per complex (Fig. 4a) despite all three signal peptide binding sites being occupied (Fig. 3b), suggesting the folded domains adopt highly variable positions rather than docking in a defined orientation.

A cryo-EM map was generated for the complex between TatBC and the most ordered of the bound MdoD proteins (Fig. 4b and Extended Data Fig. 6d). This reveals that the folded domain of the substrate does not form direct, ordered contacts with the membrane portion of the TatBC complex (Fig. 4b). The folded MdoD domain is less well resolved than the TatBC portion, probably owing to the mobility of even this most-ordered copy of the substrate protein around a flexible tether sequence (below) that links the TatBC-bound signal peptide to the folded domain of the substrate protein (Fig. 4b).

Focused refinement on the substrate density in the TatBC–MdoD complex yielded a 2.1 Å map for MdoD (Fig. 4c and Extended Data Fig. 6d). The map shows that MdoD is a tail-to-tail homodimer, consistent with a recent crystal structure^53^. Homo-oligomeric Tat substrates, such as MdoD, have a signal peptide on each protomer and thus the potential for each subunit to be independently exported. Given this possibility, it has been a long-standing question whether the Tat system transports homo-oligomeric proteins before or after oligomer formation has taken place^25^. This question is not resolved by our structural observation that TatBC can bind dimeric MdoD, because our complex was formed by in vitro reconstitution rather than through stalling substrate transport in vivo. However, we were able to show that MdoD protomers without a signal peptide can be carried to the periplasm by signal peptide-containing MdoD protomers (Fig. 4d,e). This demonstrates that MdoD dimer formation must occur in the cytoplasm before Tat transport takes place. It also shows that only one Tat signal peptide on each MdoD dimer is required for transport, a conclusion that is consistent with our structural data showing MdoD positioned so that only one of the signal peptides in the dimer can interact with the TatBC complex (Fig. 4b,e). More generally our MdoD experiments suggest that homo-oligomeric Tat substrates are transported after the constituent subunits have assembled, as is the case for hetero-oligomeric Tat substrates^2,54,55^

**Extended Data Fig. 8:**
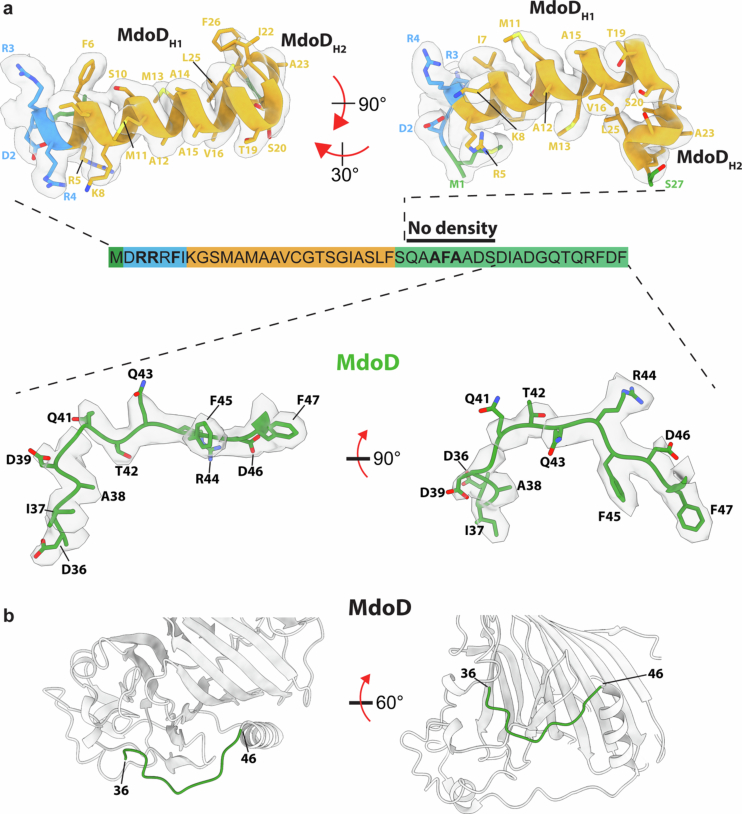
The disordered linker between the signal peptide and folded domain of MdoD extends from residue 28 to 35. **a**, The signal peptide (above) and first ten residues of the folded domain (below) are shown in their respective cryo-EM volumes (semi-transparent, grey). The region between these residues is not present in either the membrane domain-focused or folded substrate domain-focused cryo-EM volumes and is presumed disordered. (Centre) The amino acid sequence corresponding to MdoD residues 1 – 47. Residues where density is not visible in the TatBC-signal peptide volume or MdoD folded domain volume are highlighted. The protein models are coloured according to the schematic. **b**, The location of MdoD residues 36-46 (green) which pack against the rest of the folded domain of MdoD (light grey).

In our TatBC–MdoD complex volumes, the first 27 residues of the 32 residue MdoD signal peptide can be modelled before the density weakens (Fig. 3c and Extended Data Fig. 8a). MdoD density is next resolvable at residue 36, although it remains poorly ordered and with minimal contacts to the folded part of the molecule until residue 48 (Fig. 4f and Extended Data Fig. 8b). Thus, there is a tether of at least 7, and possibly as many as 19, flexible amino acids linking the bound signal peptide to the folded domain of the substrate (Fig. 4f and Extended Data Fig. 8b). Deletions extending even to the entirety of this flexible tether (residues 28–46) reduce but do not abolish the export of the substrate protein (Fig. 4g). This indicates that a stretch of unstructured protein between the signal peptide and folded domain of the substrate protein promotes efficient transport but is not essential for substrate transport to occur.

### TatA introduces asymmetry into the Tat core complex

TatA is structurally similar to TatB and can occupy the TatB binding site when TatB is absent^34^, leading to the proposal that TatB is displaced by TatA during translocation^36,37^. However, our structures show the TatB site is buried within the core complex, making exchange with TatA unlikely. To clarify how TatA interacts with the core complex, we undertook a structural analysis of TatAC complexes.

**Fig. 5:**
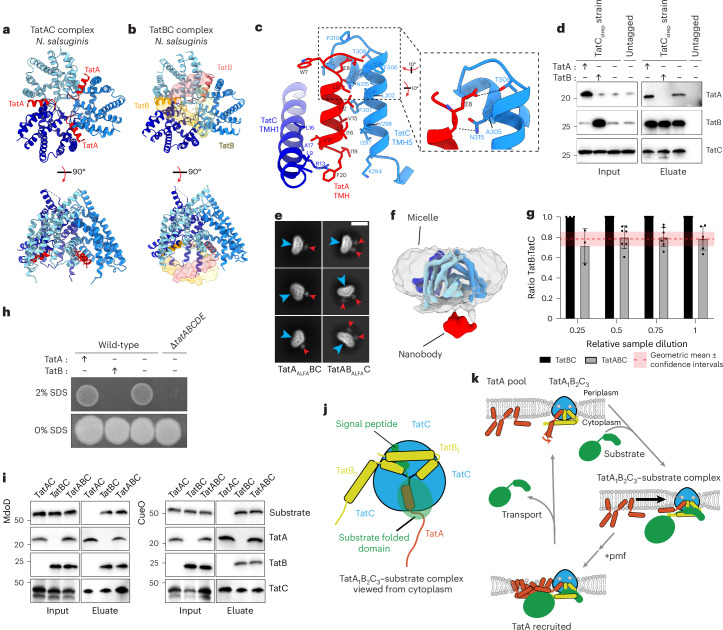
TatA is a constitutive component of the Tat receptor complex. **a**,**b**, Models of the *N. salsuginis* TatAC (**a**) and TatBC (**b**) complexes viewed from the cytoplasm (top) and the membrane plane (bottom). The TatC subunits are depicted in various shades of blue. The APH of TatB is shown as a semi-transparent surface to allow a clearer visualization of the TMH. **c**, The structure of a *N. salsuginis* TatA–TatC interface showing the interactions between the TatA TMH and TatC-TMH1 and TatC-TMH5. Residues involved in TatA–TatC contacts are labelled. Right, inset: the zoomed-in view highlights the interactions found at the previously proposed ‘polar cluster site’. Hydrogen bonds are depicted by dashed lines. **d**, A demonstration that the native *E. coli* core complex contains TatA and examination of the effects of TatA and TatB overproduction on the loading of TatA and TatB into the complex. Native core complexes isolated through modification of the TatC protein with a Twin-Strep tag and streptactin affinity chromatography are characterized by immunoblotting. The blots characterize the fully native complex (lane 3, both blots) or the complexes present when TatA or TatB are additionally overproduced (↑) from an expression plasmid (lanes 1 and 2, both blots). A wild-type (untagged TatC) strain was used as a negative control (lane 4, both blots). **e**, Representative 2D class averages of side views of the *E. coli* TatABC complexes) (blue arrows) with either TatA (LH panel) or TatB (RH panel) labelled with a nanobody (red arrows) directed against an inserted ALFA tag in either TatA or TatB, respectively. Scale bar, 100 Å. **f**, The cryo-EM volume for the nanobody-bound TatA_ALFA_BC complex viewed from the membrane plane. Density corresponding to the TatC molecules is coloured in various shades of blue (contour level 0.5), to the surrounding detergent micelle in grey and to the nanobody in red (both contoured to 0.25). **g**, The ratio of TatB to TatC in TatBC and TatABC samples quantified from Coomassie stained gels at four dilutions (total of seven repeats from three independent samples at each dilution) demonstrates that there is relatively less TatB in the TatABC complex. Geometric mean 0.78, 95% confidence interval 0.71–0.85, *P* = 0.003 (paired *t*-test, two-tailed). **h**, The effect of TatA and TatB overproduction on Tat transport. TatA or TatB were overproduced where indicated (↑) from a plasmid in either a Tat wild-type or Tat null (Δ*tatABCDE*) background and Tat function assessed through the ability to grow in the presence of SDS. **i**, Only core complexes containing TatB are able to bind Tat substrates. *N. salsuginis* TatA_His_C_strep_, TatB_FLAG_C_strep_ or TatA_His_B_FLAG_C_strep_ complexes were immobilized on streptactin affinity resin and incubated with either purified MdoD_Myc_ (left) or CueO_Myc_ (right) Tat substrates. After extensive washing, proteins were eluted and subjected to immunoblotting with antibodies against the protein epitope tags. **j**, A model for substrate binding by the Tat core complex based on the results of this study. The core complex is composed of one copy of TatA, two copies of TatB and three copies of TatC. There is one fully formed substrate binding site (that is, one that includes two TatB molecules). Substrate signal peptide binds at this site, positioning the substrate folded domain (shown in transparent rendering) at the opposite side of the complex and adjacent to the TatA site. **k**, The updated model of the Tat translocation mechanism based on the results of this study. A TatA_1_TatB_2_TatC_3_ core complex thins the membrane around the complex, whereas a large pool of TatA molecules is located in the bulk membrane. The substrate docking at the core complex (in **j**) triggers the pmf-dependent accumulation of additional copies of TatA in the vicinity of the core complex aided by the local membrane thinning, interactions with the substrate folded domain and changes in the position of the APH of the core complex TatA molecule. Concentrating the TatA molecules results in further local thinning of the membrane bilayer proximal to the substrate folded domain promoting substrate translocation across the membrane bilayer. Source data

**Extended Data Fig. 9:**
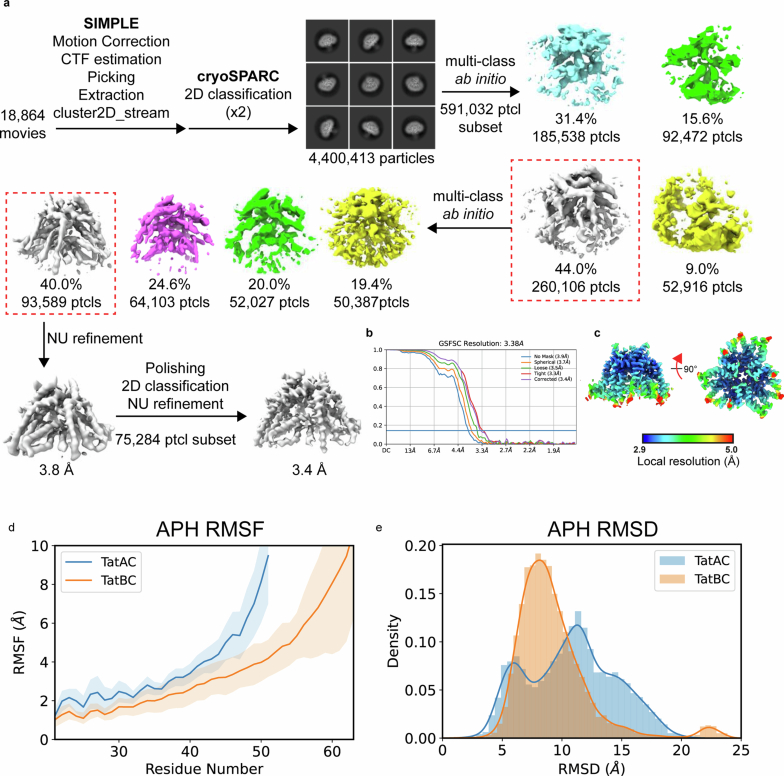
Cryo-EM workflow for the *N. salsuginis* TatAC complex and simulations of TatA complexes. **a-c**, CryoEM workflow for the *N. salsuginis* TatAC complex. **a**, Image processing workflow. **b**, Gold-standard Fourier Shell Correlation (FSC) curves used for global resolution estimation. **c**, Local resolution estimate of the volume. **d,e**, Comparative atomistic MD simulations of the E. coli TatAC and TatBC complexes in a membrane bilayer. **d**, Root Mean Square Fluctuation (RMSF) of the amphipathic helix of TatA and TatB, indicating differences in flexibility along the helix. **e**, TatA and TatB APH RMSD distributions. The RMSD and RMSF were calculated using the TatA/TatB TMH as a reference to align the MD frames. Data are based on three independent 500 ns simulations (n = 3000 MD frames). Data are presented as mean values +/- SD.

**Extended Data Fig. 10:**
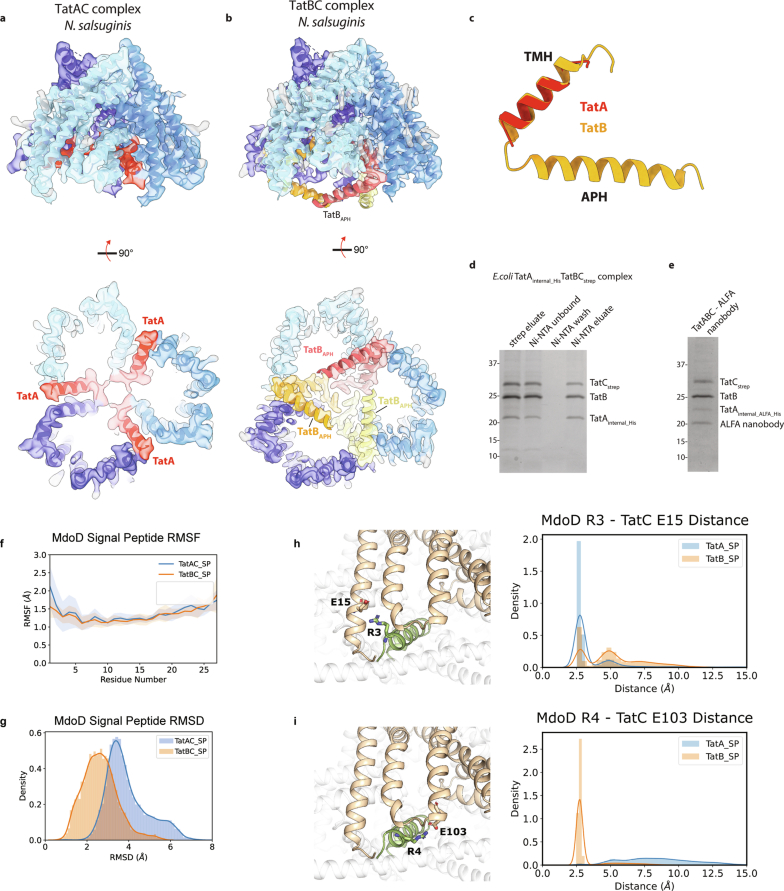
Incorporation of TatA into the Tat core complex complex and simulations of signal peptide contacts. **a**,**b**, Models of *the N. salsuginis* TatAC (**a**) and TatBC (**b**) complexes shown as cartoon representations (TatC-blue, TatA-red, TatB-yellow/orange/red) in their respective cryoEM volumes (semi-transparent) viewed from the plane of the membrane (top) or the cytoplasm (bottom). In the view from the cytoplasm, structures behind the view plane have been removed to aid interpretation. **c**, Superposition of the structurally defined portions of the TatA (red) and TatB (gold) subunits in the *N. salsuginis* TatAC and TatBC structures following alignment on the TatC subunits (Cα RMSD ~ 0.6 Å). Alignments and RMSD were calculated using the MatchMaker command in ChimeraX^85^. **d**, Isolation of an *E. coli* TatA_His_TatBC_strep_ complex solubilized in GDN and purified successively by strep tag affinity chromatography and Ni-NTA affinity chromatography. The indicated samples are analysed by SDS-PAGE and stained with Coomassie Blue. **e**, Nanobody labelling of the TatA subunit within the TatABC complex. An ALFA nanobody was mixed with purified *E. coli* TatA_ALFA,His_TatBC_strep_ complex and subject to separation by size exclusion chromatography. Coomassie Blue stained SDS-PAGE gel of the fraction from the size exclusion column used for cryoEM. **f-i**, Atomistic MD simulations of MdoD signal peptide-bound *E. coli* TatAC and TatBC complexes in a membrane environment. **f**, RMSF and **g**, RMSD distributions of the signal peptide when bound to the TatAC and TatBC complexes. RMSF and RMSD were calculated using TatC as reference for aligning the MD simulation frames. **h**, MdoD R3 – TatC E15 and **i**, MdoD R4 - TatC E103 distance distributions. Distances were defined as the minimum distance between the hydrogen bond donor/acceptor atoms. Data are derived from three independent simulations of 500 ns each (n = 3000 MD frames). Data are presented as mean values +/- SD. Source data

An *E. coli* TatAC complex structure could not be reconstructed owing to extreme compositional heterogeneity. However, we were able to determine a 3.9 Å resolution reconstruction of the *N. salsuginis* TatAC complex (Fig. 5a and Extended Data Figs. 9 and 10). In this complex, the TatA TMH occupies precisely the same site as the TatB TMH in the TatBC structures (Fig. 5b and Extended Data Fig. 10a–c), and the structure of the TatC components is unaltered within experimental error. The highly conserved polar amino acid at TatA position 8 (E8 in *N. salsuginis* TatA, corresponding to Q8 in *E. coli* TatA) makes equivalent interactions to TatB E8, whereas the hydrophobic residues in the TatA TMH make analogous packing interactions to TatB with the adjacent TatC molecules (Fig. 5c). Overall, despite sequence differences between the TMHs of TatA and TatB, all TatB TMH interactions with TatC are preserved in the TatAC complex (Fig. 5c). The most striking difference between the TatAC and TatBC structures is the lack of observable density for the TatA APH, suggesting that it is more mobile than the well-ordered APH of TatB (Extended Data Fig. 10a,b). This interpretation is supported by comparative MD simulations of the TatAC and TatBC complexes, which show that the TatA APH is highly mobile when compared with TatB (Extended Data Fig. 9d,e).

We next asked how TatA is incorporated into the full core complex containing all three Tat components. To ensure that we were structurally characterizing the physiologically relevant TatABC complex, we initially purified endogenous *E. coli* complexes using an affinity tag on the chromosomally-encoded TatC protein. Although this preparation confirmed that native Tat complexes contain TatA in addition to TatB and TatC^34^ (Fig. 5d), it produced insufficient material for cryo-EM analysis. We, therefore, moved to purifying TatABC complexes from cells expressing the *tatABC* operon from a low copy number plasmid. To assess whether all Tat core complexes contain TatA we used a dual-affinity tag strategy with distinct affinity tags on TatA and TatC, purifying sequentially, first by the tag on TatC, followed by the tag on TatA. There was no increase in the TatA-to-TatC ratio from input to eluate after the second purification step targeting TatA (Extended Data Fig. 10d), indicating that all the complexes isolated via the TatC affinity tag also contain TatA.

Cryo-EM data were collected for TatABC complexes with and without bound substrate. The highest-resolution volume obtained was from TatABC with bound CueO substrate and yielded a 3.3-Å reconstruction (Supplementary Fig. 4) that was comparable to our TatBC structures. This similarity demonstrates that incorporation of TatA does not induce any fundamental structural rearrangements within the core complex. Notably, no additional density was observed at the periphery of the complex where TatA has previously been proposed to localize^36,37^ suggesting that the TatA present occupies positions equivalent to TatB. However, the lack of side chain detail on the TMHs did not allow sites occupied by TatA or TatB TMHs to be distinguished despite extensive classifications and realignments. To visualize TatA in the 2D classes, we inserted an ALFA tag into the cytoplasmic part of TatA to allow TatA protomers within the core complex to be identified through the binding and visualization of an anti-ALFA nanobody (Supplementary Fig. 5). The 2D class averages of the nanobody-bound TatABC complex contained additional density adjacent to the core complex, which we assign to the bound nanobody (Fig. 5e). Weak density corresponding to the ALFA nanobody was also visible in the corresponding three-dimensional (3D) reconstructions (Fig. 5f and Supplementary Fig. 5). All particles from which the Tat complex could be reconstructed appeared to have a single bound nanobody (Fig. 5f), indicating that the core complex contains one TatA molecule. By contrast, the introduction of an analogous ALFA tag into TatB led to 2D class averages in which two additional densities adjacent to the core complex (Fig. 5e) could be detected, suggesting that there are two copies of TatB in this object. From these observations, we infer that the core complex has a TatA_1_B_2_C_3_ subunit stoichiometry and that a TatA molecule replaces one of the three TatB subunits found in the TatBC complex thereby forming an inherently asymmetric object. Consistent with this interpretation, we find that the ratio of TatB to TatC is significantly reduced in the TatABC complex relative to the TatBC complex (Fig. 5g).

If the three TatA/B binding sites require a fixed 1:2 TatA:TatB stoichiometry, cells would need to carefully regulate relative TatA and TatB levels. Testing this (Fig. 5d), we found that overproducing TatB reduced TatA copurification with TatC, confirming competition between TatA and TatB for the same sites. Conversely, excess TatA did not displace TatB, consistent with TatB having the higher affinity for TatC and allowing TatB to outcompete the naturally more abundant TatA under native conditions^3,56,57^.

If TatA inclusion in the core complex is functionally important, displacing it by TatB overproduction should impair transport. In agreement with this prediction, TatB overproduction blocked growth on SDS, which requires Tat-exported amidases^58^ (Fig. 5h), whereas TatA overproduction did not. Together with our structural data, this observation supports the conclusion that the functionally relevant Tat core complex is an asymmetric TatA_1_B_2_C_3_ assembly in which TatA incorporation is essential for activity.

To further probe the function of TatA relative to TatB in the core complex we compared the ability of purified *N. salsuginis* TatAC, TatBC and TatABC complexes to form stable complexes with the *E. coli* substrate proteins CueO and MdoD (no well-behaved *E. coli* TatAC complex being available for this purpose). Only those core complexes containing TatB protomers were able to bind substrate proteins (Fig. 5i). This suggests that the TatA-containing site in the TatABC complex is unlikely to be the primary site of substrate docking. This inference is supported by analysis of the TatB–signal peptide contacts, which reveals that only 4 of the 15 interactions are likely to be recapitulated by the TatA sequence (Fig. 5c and Supplementary Fig. 1c) and by MD simulations that show that TatAC complexes coordinate signal peptides less stably than TatBC complexes (Extended Data Fig. 10f–i). In the TatBC–substrate complexes, substrate binding involves wedging the substrate signal peptide between the APH of TatB and the body of the complex (Fig. 3b,d). However, as discussed above, the APH of TatA is highly mobile in the TatAC complex (Fig. 5a, Extended Data Fig. 10f–i and Supplementary Fig. 4c) and may be unable to fulfil an equivalent clamping function. This provides a possible molecular explanation for why only TatB supports tight substrate binding.

## Discussion

Here, we provide structural snapshots of the Tat core complex both in the resting state and at the earliest stage of transport when substrate protein first associates with the complex. Our structures show that the core complex recognizes substrates exclusively through their signal peptides, as previously anticipated^59^. Binding involves specific protein–protein interactions between the signal peptide n-region consensus motif and TatC residues. In addition, the signal peptide h-region is clamped by both the TMH and APH of neighbouring TatB subunits, consistent with earlier observations^24,60^. Unexpectedly, the signal peptide also contacts ordered phospholipids within the core complex, at least some of which are functionally important.

The binding of Tat signal peptides to the core complex can be compared with that of the structurally similar (although lacking a conserved sequence motif) signal peptides that target substrates to the Sec translocon. Tat signal peptides bind at the cytoplasmic face of the core complex, whereas Sec signal peptides adopt a membrane-spanning orientation bound partially within the Sec apparatus and partially exposed to the lipid bilayer^61^. Despite this difference in orientation, both signal peptide types engage lipids. Tat signal peptides interact with lipids trapped within the core complex, whereas Sec signal peptides interact with the hydrophobic bilayer interior via their h-region and with phospholipid head groups via their positively charged n-region.

In addition to characterizing the initial core complex-substrate complex, our work uncovers fundamental features of the core complex relevant to later stages in the Tat transport cycle.

Our structure of the core complex strongly disfavours earlier mechanistic models in which substrate transport occurs through its interior^11,12,62–66^. Although the core complex has a substantial internal cavity, protein transfer through its centre is highly unlikely for three reasons: the cavity is lined with hydrophobic residues and filled with lipids rather than forming an aqueous environment; the TatC subunits are tightly packed together at the periplasmic face and unlikely to open for substrate egress (and, in the *M. xanthus* complex, would additionally be blocked by the highly structured periplasmic domain; Fig. 1c), an inference supported by stability in MD simulations (Supplementary Fig. 2); and the cavity is too small to accommodate the folded domain of many Tat substrates (Supplementary Fig. 6). Thus, our structural data imply that substrate transport must occur on the periphery of the core complex as previously envisaged in some models^20,34^.

Our structures also reveal that the transmembrane regions of the Tat core complex are highly tilted, leading to extreme thinning of the surrounding membrane bilayer. We propose that this aids in the recruitment of additional copies of TatA to the core complex by relieving the hydrophobic mismatch between the short TatA TMH and the width of the bulk membrane^29^. This inference is consistent with substrate transport occurring on the periphery of the core complex (above) and supports mechanistic models that incorporate membrane thinning as critical for transport^29,44,67–70^. Emerging evidence indicates that membrane thinning is a common feature of protein translocation and membrane protein insertion systems^71^.

Finally, we find that the physiological core complex contains an unequal number of TatA and TatB subunits, each occupying an equivalent site between the TatC subunits, with our data indicating that the stoichiometry is likely TatA_1_TatB_2_TatC_3_. The result is that the physiological core complex is inherently asymmetric. As our biochemical studies indicate only TatB, and not TatA, is able to support substrate binding to the core complex, and our substrate-bound structures show that two copies of TatB are needed to create one signal peptide-binding site, we infer that there is only a single physiologically relevant signal peptide-binding site in a TatA_1_B_2_C_3_ complex (because there is only one TatC subunit for which signal peptide binding is assisted by two copies of TatB) (Fig. 5i). This signal peptide binding site is positioned across the complex from the TatA molecule (Fig. 5i). Consequently, the orientation of the bound signal peptide across the centre of the core complex seen in this work will direct the attached substrate folded domain towards the TatA site (Fig. 5i). We therefore speculate that the asymmetry of the core complex establishes a mode of substrate binding in which the folded domain is positioned for transport at the TatA side of the core complex (Fig. 5i) with this localization promoting the clustering of additional TatA molecules to enable transport. Further work will be required to test this model.

## Methods

### Bacterial strains and plasmids

Bacterial strains and plasmids used in this study are listed in Supplementary Table 1. The genes encoding *M. xanthus* (ATCC 19368) TatB and TatC and *N. salsuginis* (strain E9I37-1T) TatA, TatB and TatC were codon-optimized for expression in *E. coli* and synthesized as DNA fragments containing homologous recombination sites for Gibson assembly. PCR fragments for *E. coli tatA*, *tatB*, *tatC*, *cueO* and *mdoD* were generated by PCR using Q5 polymerase (NEB) and *E. coli* genomic DNA (DSM 1116). Gibson assembly and PCR reactions were carried out according to the manufacturer’s recommendations.

### Chromosomal modifications

Gene deletions, point mutations and the insertion of sequences encoding Twin-Strep tags in the bacterial chromosome, were performed using the pWRG730 and pWRG717 constructs, using a modified protocol based on Hoffmann et al.^72^. In brief, an aphI-I-SceI kanamycin resistance cassette was amplified from pWRG717 with primers that incorporate homology regions to the target gene. *E. coli* strains carrying the temperature-sensitive construct pWRG730 were grown at 30 °C to and absorbance at 600 nm (A_600nm_) = 0.4–0.6 in lysogeny broth (LB; 0.5% w/v yeast extract, 1% w/v tryptone, 170 mM NaCl). The λ red recombinase expression was induced by incubating cultures in a 42 °C water bath for 13 min; cultures were subsequently incubated on ice for 20 min. Cells were harvested by centrifugation and washed three times in ice-cold water. Cells were resuspended in 100 μl of ice-cold water, and 100 ng of PCR product was added. Cells were electroporated and recovered in 0.5 ml of SOC media (0.5% w/v yeast extract, 2% w/v tryptone, 10 mM NaCl, 2.5 mM KCl, 10 mM MgCl2, 10 mM MgSO4, 20 mM glucose) at 30 °C for 1 h. Colonies were selected on LB agar containing kanamycin (50 μg ml^−1^) and chloramphenicol (10 μg ml^−1^). To introduce point mutations or tags onto the chromosome, a second PCR product was generated that incorporated the point mutation or tag and at least 400 bp flanking the gene of interest. The λ red recombinase expression in strains carrying the aphI-I-SceI kanamycin resistance cassette was carried as described above and electroporated with 500–1,000 ng of the PCR product containing the point mutations or tag. Cells were selected on LB agar plates containing chloramphenicol (10 μg ml^−1^) and anhydrotetracycline (500 ng μl^−1^). Positive clones were verified by PCR and DNA sequencing.

### Purification of *M. xanthus* and *N. salsuginis* TatBC and TatAC complexes

*M. xanthus* and *N. salsuginis* TatBC complexes or the *N. salsuginis* TatAC complex were overexpressed in *E. coli* strain L56 carrying both pTatC-GFP-His (pWALDO-based) and pTatB-strep (pCDFDuet-1-based) or pTatAstrep (pCDFDuet-1-based). Cells were grown in terrific broth (TB, 2.4% w/v yeast extract, 1.2% w/v tryptone, 0.4% w/v glycerol, 17 mM KH_2_PO_4_, 72 mM K_2_HPO_4_) containing spectinomycin (100 μg ml^−1^) and kanamycin (50 μg ml^−1^) at 37 °C with shaking to A_600nm_ = 4.0 after which cultures were supplemented with 0.1 mM isopropyl β-D-1-thiogalactopyranoside (IPTG) and incubated at 25 °C for an additional 12 h with shaking. Cells were harvested by centrifugation (5,000*g*, 10 min). Purification steps were similar for *M. xanthus* and *N. salsuginus* constructs and carried out at 4 °C. In brief, cell pellets were resuspended in PBS (10 mM Na_2_HPO_4_, 1.8 mM KH_2_PO_4_, 2.7 mM KCl, 137 mM NaCl) supplemented with DNase I (30 μg ml^−1^), lysozyme (500 μg ml^−1^) and a cOmplete EDTA-free Protease Inhibitor Cocktail tablet (Sigma) for 30 min before passing through an EmulsiFlex C5 homogenizer (Avestin) at 15,000 psi. Lysates were clarified by centrifugation (30,000*g*, 30 min) and membranes were collected by centrifugation (200,000*g* for 1.5 h). Membranes were resuspended in buffer A (50 mM sodium phosphate pH 8.0, 300 mM NaCl) containing 20 mM imidazole and solubilized by incubation with 1% (w/v) glyco-diosgenin (GDN; Anatrace) for 2 h. Insoluble material was removed by centrifugation (100,000 *g*, 30 min) and solubilized membranes applied to three 5-ml Ni-NTA superflow cartridges (QIAGEN). The resin was washed with 10 column volumes (10 CV) of buffer A containing 0.02% (w/v) GDN and 20 mM imidazole, followed by washing with 10 CV of buffer A containing 0.02% (w/v) GDN and 40 mM imidazole. Proteins were eluted in 4 CV of buffer A containing 0.02% (w/v) GDN and 300 mM imidazole. TEV protease was added to the eluates at a ratio of TEV to TatBC or TatAC of 1:100 and the sample dialysed overnight using 10,000 molecular weight cut-off (MWCO) SnakeSkin dialysis tubing (Thermo Scientific) into buffer B (50 mM sodium phosphate 8.0, 150 mM NaCl, 0.02% GDN). The dialysed sample was adjusted to contain 20 mM imidazole and passed through a 5-ml Ni-NTA superflow cartridge (QIAGEN) to remove GFP–His. The sample was then applied to a 5-ml Streptactin XT superflow column (IBA Lifesciences). The resin was washed with 20 CV of buffer B (50 mM sodium phosphate pH 8.0, 150 mM NaCl, 0.5 mM EDTA) containing 0.02% GDN, and the proteins were eluted in 5 CV of buffer B supplemented with 50 mM D-biotin (IBA Lifesciences) and 0.02% GDN. Eluates were concentrated using a 100 kDa MWCO Vivaspin 20 (cytiva) centrifugal filter unit and injected onto a Superose 6 Increase 10/300 GL size exclusion column (cytiva) pre-equilibrated in buffer C (20 mM HEPES pH 7.4, 150 mM NaCl, 0.02% GDN). Peak fractions were collected and concentrated using a 100 kDa MWCO Vivaspin (GE Healthcare) centrifugal filter unit.

### Purification of *E. coli* TatBC and TatABC complexes

*E. coli* TatBC and TatABC complexes were overexpressed in an *E. coli* MC4100 Δ*tatABCD*, Δ*tatE* carrying pQE60-based vectors and pREP4 (listed in Supplementary Table 1). Cultures were grown in LB supplemented with 4% glycerol, kanamycin (50 μg ml^−1^) plus carbenicillin (100 μg ml^−1^) or chloramphenicol (25 μg ml^−1^) at 37 °C with shaking to A_600nm_ = 0.7. Cells were then supplemented with 1 mM IPTG and incubated at 37 °C for an additional 4 h with shaking. Cell pellets were resuspended in lysis buffer (50 mM Tris pH 8.0, 200 mM NaCl, 1 mM EDTA) supplemented with DNase I (30 μg ml^−1^), lysozyme (500 μg ml^−1^) and a cOmplete EDTA-free Protease Inhibitor Cocktail tablet (Sigma) for 30 min before passing through an EmulsiFlex C5 homogenizer (Avestin) at 15,000 psi. Lysates were clarified by centrifugation (12,000*g*, 10 min) and membranes were collected by centrifugation (150,000*g*, 1.5 h). Membranes were resuspended in lysis buffer and solubilized by incubation with 1% (w/v) GDN (Anatrace) at 4 °C for 16 h. Insoluble material was removed by centrifugation (150,000*g*, 40 min) and solubilized membranes were supplemented with 12 mM imidazole then mixed with pre-equilibrated cOmplete His tag purification resin (Roche) for 3 h at 4 °C. The resin was centrifuged at 500*g* for 5 min and then transferred to a gravity column. The resin was washed with 15 CV of lysis buffer supplemented with 12 mM imidazole and 0.02% GDN, followed by elution in lysis buffer supplemented with 100 mM imidazole and 0.02% GDN. Eluates were concentrated using a 100 kDa MWCO Vivaspin 20 (cytiva) centrifugal filter unit and injected onto a Superose 6 Increase 10/300 GL size exclusion column (cytiva) pre-equilibrated in lysis buffer plus 0.02 % GDN. Peak fractions were collected and concentrated using a 100 kDa MWCO Vivaspin (GE Healthcare) centrifugal filter unit.

Membranes from cells overexpressing *E. coli* TatABC complexes containing an internal His tag or ALFA-His tag in TatA or TatB and a C-terminal strep tag in TatC were prepared and solubilized with GDN as described above for the *E. coli* TatBC complex. Insoluble material was removed by centrifugation (100,000*g*, 30 min) and solubilized membranes applied to a 5-ml Streptactin XT superflow column (IBA Lifesciences). The resin was washed with 20 CV of buffer B (50 mM sodium phosphate pH 8.0, 150 mM NaCl, 0.5 mM EDTA) containing 0.02% GDN and proteins eluted in 5 CV of buffer B supplemented with 50 mM D-biotin (IBA Lifesciences) and 0.02% GDN. The sample was adjusted to containing 3 mM imidazole and passed through a cOmplete His tag purification column (Roche). The column was washed with 20 CV of buffer A containing 0.02% (w/v) GDN and 2 mM imidazole. Proteins were eluted in 6 CV of buffer A containing 0.02% (w/v) GDN and 200 mM imidazole. Eluates were concentrated using a 100 kDa MWCO Vivaspin 20 (cytiva) centrifugal filter unit and injected onto a Superose 6 Increase 10/300 GL size exclusion column (cytiva) pre-equilibrated in buffer C (20 mM HEPES pH 7.4, 150 mM NaCl, 0.02% GDN). Peak fractions were collected and concentrated using a 100 kDa MWCO Vivaspin (GE Healthcare) centrifugal filter unit.

### Purification of selenomethionine-labelled *M. xanthus* TatC periplasmic domain

The periplasmic domain (incorporating residues 127-303) of *M. xanthus tatC* was subcloned into a pET15b vector by PCR amplification of codon-optimized *M. xanthus tatC*, followed by restriction digestion and ligation. Protein was overexpressed in the auxotrophic *E. coli* strain B834 (DE3) grown in SelenoMethionine media (Molecular Dimensions) plus 100 μg ml^−1^ carbenicillin after induction of A_600nm_ = 0.6 cultures with 1 mM IPTG at 21 °C for 18 h with shaking. Cells were harvested by centrifugation (5,000*g*, 10 min). Cell pellets were resuspended in lysis buffer containing 20 mM HEPES pH 7.5, 300 mM NaCl, 10 mM imidazole, 2 mM TCEP, and supplemented with DNase I (30 μg ml^−1^), lysozyme (500 μg ml^−1^) and a cOmplete EDTA-free Protease Inhibitor Cocktail tablet (Sigma) for 30 min before passing through an EmulsiFlex C5 homogenizer (Avestin) at 15,000 psi. Lysates were clarified by centrifugation (30,000 *g*, 30 min), and the supernatant was applied to a 5-ml Ni-NTA superflow cartridge (Qiagen) pre-equilibrated with lysis buffer. The resin was washed with 10 CV of lysis buffer followed by 20 CV of lysis buffer supplemented with 20 mM imidazole. The protein was eluted in lysis buffer supplemented with 250 mM imidazole followed by addition of TEV protease (1:100 ratio of TEV to TatC periplasmic domain), and the sample was then dialysed overnight using 3,000 MWCO SnakeSkin dialysis tubing (Thermo Scientific) into 20 mM HEPES pH 7.5, 150 mM NaCl and 2 mM TCEP. The dialysed sample was adjusted to contain 20 mM imidazole and passed through a 5-ml Ni-NTA superflow cartridge (QIAGEN) to separate the proteolyzed His_6_-tag and His-tagged TEV protease from the flow-through. The flow-through was subsequently concentrated using a 10 kDa MWCO Vivaspin 20 (cytiva) centrifugal filter and injected onto a Superdex 75 26/600 size exclusion column (cytiva) pre-equilibrated in buffer containing 20 mM HEPES pH 7.5, 150 mM NaCl and 2 mM TCEP. Peak fractions were collected and concentrated to an *A*_280nm_ of 8.9 using a 10 kDa MWCO Vivaspin (cytiva) centrifugal filter unit for crystallization.

### Crystallization and data collection and processing

Crystallization was carried out using the sitting-drop vapour diffusion method at 21 °C. Crystals were obtained in 0.2 M lithium sulphate, 0.1 M Tris pH 8.5, 10% PEG 8000 and 10% PEG 1000 at a 1:1 ratio of protein:mother liquor and a drop volume of 400 nl. These were flash frozen in liquid nitrogen in the mother liquor supplemented with 25% ethylene glycol. Diffraction data were collected to 2.0 Å at the Diamond light source (Beamline I02), at 120 K from 1 SeMet-labelled crystal (*λ* = 0.97926). Data were indexed, integrated and scaled using iMosflm^73^ and point and scale within CCP4. In total, 18 SeMet sites were detected using Phenix Autosol^74^ with a FOM of 0.327. The data were phased, the density was modified and the initial model was built using Phenix Resolve^75^ (map skew 0.3, correlation of local root mean square density 0.87). The model was iteratively manually adjusted in Coot^76^ and refined in Phenix^77^ to produce the model described in Supplementary Table 2.

### Purification of TatABC– and TatBC–substrate complexes

Full-length MdoD or CueO was expressed in *E. coli* strain BL21 (DE3) Δ*tatABCD* and Δ*tatE* from a pET28a vector modified to encode an N-terminal His_6_-SUMO fusion. Cells were grown in LB media containing kanamycin (50 μg ml^−1^) at 37 °C with shaking to A_600nm_ = 0.8; after which, cultures were supplemented with 1 mM IPTG and incubated at 18 °C for an additional 12 h with shaking. Cells were harvested by centrifugation (5,000 *g*, 10 min). Cell pellets were resuspended in Buffer D (50 mM Tris.HCl pH 8.0, 500 mM NaCl, 1 mM EDTA and 2 mM imidazole) supplemented with DNase I (30 μg ml^−1^), lysozyme (500 μg ml^−1^) and a cOmplete EDTA-free Protease Inhibitor Cocktail tablet (Sigma) for 30 min before passing through an EmulsiFlex C5 homogenizer (Avestin) at 15,000 psi. Lysates were clarified by centrifugation (30,000*g*, 40 min) and applied to a 1 ml cOmplete His tag purification column (Roche) and the resin washed with 40 CV of buffer D. Proteins were eluted in 6 CV of buffer E (50 mM Tris–HCl pH 8.0, 150 mM NaCl, 1 mM EDTA, 200 mM imidazole) and the eluate dialysed into 20 mM HEPES pH 7.4, 150 mM NaCl and 1 mM EDTA using 10,000 MWCO SnakeSkin dialysis tubing (Thermo Scientific). To assemble the TatBC–MdoD and TatABC–CueO complexes, His_6_-SUMO-MdoD or His_6_-SUMO-CueO were incubated at 4 °C for 1 h with *Saccharomyces cerevisiae* Ubiquitin-like-specific protease 1 (Ulp1), the buffer adjusted to contain 0.02% GDN and the samples mixed with purified *E. coli* TatBC or TatABC complex. The samples were incubated overnight at 4 °C and injected onto a Superose 6 Increase 10/300 GL size exclusion column (cytiva) pre-equilibrated in 50 mM Tris pH 8.0, 200 mM NaCl, 1 mM EDTA and 0.02% GDN. Peak fractions were collected and concentrated using a 100 kDa MWCO Vivaspin (GE Healthcare) centrifugal filter unit. To assemble the *N. salsuginis* TatBC–CueO signal peptide complex, 50 μl TatBC (*A*_280nm_ = 8.0) was mixed with 50 μl CueO peptide (1 mg ml^−1^, Peptide Protein Research, residues 1–28) and incubated for 12 h at 4 °C before grid preparation.

### Reconstitution of *N. salsuginis* TatBC into MSPE3D1 nanodiscs

Chloroform was removed from *E. coli* polar lipids (Avanti) using a rotary evaporator. The lipids were washed twice with pentane and then resuspended at 20 mg ml^−1^ in MSP buffer (20 mM Tris–HCl pH 7.4, 100 mM NaCl, 1 mM EDTA) containing 60 mM cholate and stored at −80 °C until required. *N. salsuginis* TatBC was purified as described above, except membranes were solubilized in 1% digitonin instead of GDN, and purification buffers contained 0.1% digitonin rather than 0.02% GDN. For nanodisc reconstitution, digitonin-purified TatBC was mixed with membrane scaffold protein (MSP1E3D1, purified as previously described^78^) and *E. coli* polar lipids using a 1:2:122 (TatBC:MSP1E3D1:lipid mixture) molar ratio and diluted with MSP buffer to a final cholate concentration of 25 mM. The reaction mixture was incubated on ice for 1 h, followed by detergent removal using 1 g of prewashed Biobeads SM-2 (Bio-Rad) per millilitre of reaction mixture by incubation overnight at 4 °C. To enrich for nanodiscs containing TatBC, the nanodisc reaction mixture was applied to a 1-ml Streptactin XT superflow column (IBA Lifesciences), the resin washed with 20 CV of MSP buffer and proteins eluted with MSP buffer supplemented with 50 mM D-biotin (IBA Lifesciences). The eluate was concentrated using a 100 kDa MWCO Vivaspin 20 (cytvia) centrifugal filter unit and injected onto a Superose 6 Increase 10/300 GL size exclusion column (cytvia) pre-equilibrated in MSP buffer. Peak fractions were collected and concentrated using a 100 kDa MWCO Vivaspin (GE healthcare) centrifugal filter unit.

### Cryo-EM sample preparation and imaging

Purified complexes (4 μl each; preparations summarized in Supplementary Table 3) of *E. coli* TatBC (*A*_280nm_ = 4.2), *M. xanthus* TatBC (*A*_280nm_ = 4.4), *N. salsuginis* TatBC (*A*_280nm_ = 4.0), *N. salsuginis* TatBC in nanodisc (*A*_280nm_ = 2.5), *N. salsuginis* TatBC–CueO peptide (*A*_280nm_ = 4.0), *E. coli* TatABC–CueO (*A*_280nm_ = 4.3), *E. coli* TatBC–MdoD (*A*_280nm_ = 5.0), *E. coli* TatA_ALFA_BC–ALFA nanobody complex or *N. salsuginis* TatAC (*A*_280nm_ = 4.0), *E. coli* TatAB_ALFA_C–ALFA nanobody complex (*A*_280nm_ = 5.5) were adsorbed to glow discharged holey carbon-coated grids (Quantifoil 300 mesh, Au R1.2/1.3 for 15 s at 15 mA). Grids were then blotted for 2 s at 100% humidity at 4 °C and frozen in liquid ethane using a Vitrobot Mark IV (FEI). Data were collected in counted mode on a Titan Krios G3 (FEI) operating at 300 kV with a GIF energy filter (Gatan) with slit width of 20 eV at 105,000× magnification and K3 Summit detector (Gatan) or on a CFEG-equipped Titan Krios G4 (Thermo Scientific) operating at 300 kV with a Selectris X imaging filter (Thermo Fisher Scientific) with slit width of 10 eV at 165,000× magnification on a Falcon 4 direct detection camera (Thermo Fisher Scientific). Pixel sizes ranged from 0.693 Å to 0.832 Å, and movies were collected at a total dose ranging from 51.1 e^− ^A^−2^ to 62.9 e^− ^A^−2^ fractionated to 0.9–1.5 e^− ^A^−2 ^fraction^−1^ for motion correction across the various datasets (Supplementary Table 4).

### Cryo-EM data processing

Patched motion correction, contrast transfer function (CTF) parameter estimation, particle picking, extraction and initial 2D classification were performed in SIMPLE 3.0^79^. All downstream processing was carried out in cryoSPARC^80^ or RELION^81^, using the csparc2star.py script within UCSF pyem^82^ to convert between formats. Global resolution was estimated from gold-standard Fourier shell correlations using the 0.143 criterion, and local resolution estimation was calculated within cryoSPARC using a Fourier shell correlation threshold of 0.5 or within RELION^81^.

The cryo-EM processing workflow for *E. coli* TatBC in GDN is outlined in Extended Data Fig. 2a–c. In brief, particles were subjected to two rounds of reference-free 2D classification (*k* = 300) using a 160-Å soft circular mask within cryoSPARC. Four volumes were then generated from a 393,435 particle subset of the 2D-cleaned particles after multi-class ab initio reconstruction (C3 symmetry) using a maximum resolution cut-off of 6 Å. Output volumes were lowpass-filtered to 8 Å and used as references for a four-class heterogeneous refinement (C3 symmetry) against the full 2D-cleaned particle set (1,218,611 particles). Particles (468,840) from the most populated and structured class were selected and non-uniform refined, applying C3 symmetry, against their corresponding volume lowpass-filtered to 15 Å, generating a 3.2-Å map. Bayesian polishing was performed in RELION followed by local and global CTF refinement (fitting beamtilt and trefoil) and non-uniform refinement in cryoSPARC to yield a 3.1-Å map that was used for model building.

The cryo-EM processing workflow for *N. salsuginis* TatBC in GDN is outlined in Extended Data Fig. 2d–f. In brief, particles were subjected to two rounds of reference-free 2D classification (*k* = 200) using a 150-Å soft circular mask within cryoSPARC. Selected particles (1,169,012) were then input into multi-class ab initio reconstruction (C1 symmetry) using a maximum resolution cut-off of 5 Å, generating four volumes. Particles (471,101) belonging to the most populated and structured class were selected and non-uniform refined, applying C3 symmetry, against their corresponding volume lowpass-filtered to 8 Å, generating a 3.0-Å map. Bayesian polishing was performed in RELION, followed by an additional round of 2D classification in cryoSPARC (150-Å circular mask, *k* = 200) whereby 371,162 particles were kept for further processing. These particles were non-uniform refined (C3 symmetry) against an 8-Å lowpass-filtered reference, generating a 2.6-Å volume that was further improved to 2.5 Å after local and global CTF refinement (fitting beamtilt and trefoil).

The cryo-EM processing workflow for *M. xanthus* TatBC in GDN is outlined in Extended Data Fig. 3. In brief, particles were subjected to one round of reference-free 2D classification (*k* = 300) using a 150-Å soft circular mask within cryoSPARC. Three volumes were then generated from a 33,176 particle subset of the 2D-cleaned particles after multi-class ab initio reconstruction (C1 symmetry) using a maximum resolution cut-off of 5 Å. Output volumes were lowpass-filtered to 8 Å and used as references for a three-class heterogeneous refinement (C1 symmetry) against the full 2D-cleaned particle set (1,271,712 particles). Particles (636,367) from the most populated and structured class were selected and non-uniform refined, applying C3 symmetry, against their corresponding volume lowpass-filtered to 8 Å, generating a 3.3-Å map. Bayesian polishing was performed in RELION followed an additional round of 2D classification in cryoSPARC (150 Å circular mask, *k* = 200). The 2D-selected particles (530,874) were subjected to non-uniform refinement with C3 symmetry against the prepolished volume lowpass-filtered to 25 Å, yielding a 3.0-Å reconstruction. Focused alignment-free 3D classification (*k* = 8, *T* = 4, 25 iterations) using a soft mask covering TatBC TMHs was then performed, yielding two classes with improved density for the TMH of TatB. Particles belonging to both classes were combined and non-uniform refined (C3 symmetry) against a 15-Å lowpass-filtered reference, followed by local and global CTF refinement (fitting beamtilt and trefoil), to yield a 3.2-Å map that was used for model building.

The cryo-EM processing workflow for *N. salsuginis* TatBC nanodisc complex is outlined in Extended Data Fig. 5. A total of 10,945,816 particles were subjected to reference-free 2D classification in cryoSPARC (300 classes). Selected particles (1,114,288) were used to generate an ab initio model. This model was lowpass-filtered to 35 Å and used as a reference for a non-uniform refinement using an initial lowpass filter of 8 Å, to yield a 3.7-Å map. Non-uniform refinement in cryoSPARC after Bayesian polishing in RELION generated a final map with a global resolution of 3.1 Å.

The cryo-EM processing workflow for *E. coli* TatBC–MdoD in GDN is outlined in Extended Data Fig. 6a–c. Two datasets were collected for this sample. For dataset 1, particles were subjected to three rounds of reference-free 2D classification (*k* = 300) using a 180-Å soft circular mask for the first two rounds. Five volumes were then generated from a 138,750 particle subset of the 2D-cleaned particles after multi-class ab initio reconstruction (C1 symmetry) and using a maximum resolution cut-off of 7 Å. Output volumes were lowpass-filtered to 8 Å and used as references for a five-class heterogeneous refinement (C1 symmetry) against the full 2D-cleaned particle set (1,796,522 particles). Particles (350,493) belonging to the class that demonstrated structured TMHs were selected and non-uniform refined, applying C1 symmetry, against their corresponding volume lowpass-filtered to 15 Å, generating a 3.4-Å map. No asymmetry was seen in this volume, and there was equivalent occupancy of signal peptides in the three sites. Non-uniform refinement using C3 symmetry further improved map resolution to 3.0 Å. For dataset 2, particles were subjected to two rounds of reference-free 2D classification (*k* = 300) using a 180-Å soft circular mask for the first classification job. The 2D-cleaned particles (2,834,346) were then subjected to heterogeneous refinement (C1 symmetry) against the same 8-Å lowpass-filtered references generated from ab initio reconstructions described above for dataset 1. Particles (850,952) belonging to the class that demonstrated structured TMHs were selected and again showed no asymmetry and equivalent occupancy of the signal peptides in all three sites. This volume was now non-uniform refined, applying C3 symmetry, against the C1 volume lowpass-filtered to 15 Å, generating a 2.8-Å C3 volume. These particles, along with the 350,493 particles that were curated from dataset 1, were polished in RELION, combined and non-uniform refined (C3 symmetry) against a 15-Å lowpass-filtered volume to generate the 2.4 Å volume used in model building and are displayed in Figs. 2h, 3b–i and 4a,b. Local and global CTF refinement was performed but did not appreciably improve map quality. Although the input material for this dataset was generated by purification of complexes using a TatC Twin-Strep tag from overexpressed TatABC with added MdoD, we were unable to identify density corresponding to TatA in any of our reconstructions, even after extensive classification schemes targeting the signal peptide, substrate, and subunits of the Tat complex. Further analysis of the input material (there was no tag on the TatA, and it was purified solely via a tag on TatC), revealed only very low levels of TatA incorporation in the purified material. We therefore refer to this complex as TatBC–MdoD throughout.

The cryo-EM processing workflow for the *E. coli* MdoD substrate is outlined in Extended Data Fig. 6d–f. The *E. coli* MdoD volume was generated by selecting 2D class averages that corresponded to the *E. coli* mdoD substrate in 2D classifications from datasets 1 and 2 of the *E. coli* TatBC–MdoD in GDN sample. The selected 2D class averages from dataset 1 and dataset 2 were used as input for two separate multi-class ab initio reconstructions (C1 symmetry) and using a maximum resolution cut-off of 7 Å. Particles from selected classes from dataset 1 were non-uniform refined, applying C2 symmetry, using a volume lowpass-filtered to 15 Å, generating a 2.8-Å volume. The same procedure was particles from dataset 2, which also generated a 2.8 Å volume. Volumes generated from non-uniform refinements applying C1 symmetry were identical to the C2 volumes. Particles were polished in RELION, combined and non-uniform refined (C2 symmetry) against a 15-Å lowpass-filtered volume to generate the 2.3-Å volume. Local and global CTF refinement was performed, followed by a non-uniform refinement, which generated a 2.1-Å volume used in model building.

The cryo-EM processing workflow for the *N. salsuginis* TatBC–CueO peptide complex is outlined in Supplementary Fig. 3. In total, 8,504,721 particles were subjected to reference-free 2D classification in cryoSPARC (300 classes). Selected particles (1,426,112) were subjected to a multi-class heterorefinement in cryoSPARC using four 8-Å lowpass-filtered ab initio models generated from a previous data collection of a TatBC–CueO peptide sample as input, which generated a map with a resolution of 4.2 Å. A 2D classification and a non-uniform refinement in cryoSPARC after Bayesian polishing in RELION improved map quality and generated a final map with a global resolution of 3.2 Å. A local CTF refinement followed by a non-uniform refinement generated a map with a resolution of 3.1 Å. Focused 3D classification without alignment was performed in RELION using a soft mask encompassing the peptide binding cavity from which one highly occupied class (84,834 particles) was selected. This particle set was subjected to non-uniform refinement in cryoSPARC to generate a 3.2-Å volume with strong CueO peptide density.

The cryo-EM processing workflow for the *N. salsuginis* TatAC complex is outlined in Extended Data Fig. 7. In total, 12,432,717 particles were subjected to reference-free 2D classification in cryoSPARC (*k* = 300). Selected particles (591,032) were used to generate multi-class ab initio models which were lowpass-filtered to 35 Å. The particles from the selected model were used as input for a second multi-class ab initio reconstruction, which yielded a class containing 93,589 particles. This model was lowpass-filtered to 35 Å and used as a reference for a non-uniform refinement using an initial lowpass filter of 8 Å, to yield a 3.8-Å map. A 2D classification and non-uniform refinement in cryoSPARC after Bayesian polishing in RELION generated a final map with a global resolution of 3.4 Å.

The cryo-EM processing workflow for the *E. coli* TatABC–CueO complex is outlined in Supplementary Fig. 4. In brief, 5,301,381 particles were subjected to two rounds of reference-free 2D classification in cryoSPARC (*k* = 200). Five volumes were then generated from 855,150 2D-cleaned particles after multi-class ab initio reconstruction (C1 symmetry) using a maximum resolution cut-off of 7 Å. Output volumes were lowpass-filtered to 7 Å and used as references for a five-class heterogeneous refinement (C1 symmetry) against the same 855,150 2D-cleaned particles. Particles (247,806) from the most populated and structured class were selected and non-uniform refined in C1 against their corresponding volume lowpass-filtered to 15 Å, resulting in a 3.3-Å volume. Extensive classifications schemes were performed on the subunits of the Tat complex and/or signal peptide, but we were unable to resolve any inherent asymmetry.

The cryo-EM processing workflow for the *E. coli* TatA_ALFA_BC–ALFA nanobody complex is outlined in Supplementary Fig. 5. A total of 6,808,766 particles were subjected to reference-free 2D classification in cryoSPARC (*k* = 200). Selected particles (3,647,437) were subjected to a multi-class heterorefinement in cryoSPARC using four 8-Å lowpass-filtered ab initio models generated from a subset of particles as input. The particles from the selected class were used as input for another round of 2D classification; selected particles were used as input for two additonal multi-class ab initio reconstructions, which yielded a class containing 113,148 particles. This model was lowpass-filtered to 35 Å and used as a reference for a non-uniform refinement using an initial lowpass filter of 8 Å, to yield a 6.4-Å map.

Selected 2D class averages of the *E. coli* TatAB_ALFA_C–ALFA nanobody complex were obtained following two rounds of 2D classification (*k* = 200) of a 7,448,890-particle set, a multi-class ab initio (*k* = 4) reconstruction to further remove junk particles and a subsequent 2D classification (*k* = 100) on 303,279 selected particles.

Supplementary Table 3 summarizes which dataset gave rise to the volumes displayed in each figure panel.

### Cryo-EM model building and refinement

Atomic models were built into their respective cryo-EM volumes in Coot^76^. Models were further refined in real-space using PHENIX^77^ with rotamer, Ramachandran restraints and secondary structure restraints (where necessary) against either global B-factor sharpened maps or deepEMhancer maps, yielding the models described in Supplementary Table 4. All models were validated using MolProbity within PHENIX^83^. A homology model of *E. coli* MdoD was generated by sequence threading against the *E. coli* MdoG model using Phyre2^84^. Figures were prepared using UCSF ChimeraX^85^ and Adobe Illustrator.

### SDS sensitivity assay

*E. coli* strains were grown aerobically overnight at 37 °C in LB. Cultures were diluted in fresh LB grown to A_600nm_ = 1.0. For strains carrying pQE-based vectors, media and plates were supplemented with carbenicillin (100 μg ml^−1^) and IPTG (100 μM–1 mM). Cultures were diluted 1,000-fold in PBS and 5 μl spotted onto LB agar supplemented with 2% SDS and incubated overnight at 37 °C. The data presented are representative of at least three independent experiments.

### MdoD export assays

Overnight cultures of cells freshly transformed with plasmid(s) were diluted 1:40 into fresh LB media supplemented with appropriate antibiotic(s) as indicated below. For the export assays of MdoD linker variants, cultures were grown in LB containing ampicillin (100 μg ml^−1^) at 37 °C for 45, IPTG was added to a final concentration of 1 mM and cultures were grown for a further 45 min. For experiments using the MdoD signal peptide deletion construct, cultures were grown in LB with 1 mM IPTG at 37 °C for 45 min, L-arabinose was added to a final concentration of 0.2% L-arabinose and cultures were grown for a further 45 min. Cells were harvested by centrifugation (3,250*g*, 4 °C, 10 min) and resuspended in 10 mM Tris–HCl, 150 mM NaCl, pH 7.3 with cell densities normalized according to A_600nm_. Equal volumes of the resuspended cells were collected by centrifugation (16,000*g*, 4 °C, 1 min) and resuspended in 400 μl SET buffer (17% sucrose (w/v), 3 mM EDTA, 10 mM Tris–HCl pH 7.3). A total of 133 μl of lysozyme (3 mg ml^−1^) and 400 μl of ice-cold water were added and samples incubated at 37 °C for 20 min. Spheroplasts were separated from the periplasmic contents by centrifugation (16,000*g*, 4 °C, 1 min). Spheroplasts were washed in 10 mM Tris–HCl, 150 mM NaCl, pH 7.3 and collected by centrifugation (16,000*g*, 4 °C, 1 min). Samples were analysed by immunoblotting for MdoD in the linker deletion experiments using monoclonal anti-Myc antibody (antibodies are defined in Supplementary Table 5). Samples were analysed by immunoblotting for MdoD in the signal peptide deletion experiments using monoclonal anti-FLAG antibody (full-length C-terminally FLAG-tagged MdoD (MdoD_wild-type_)) or using monoclonal anti-Myc antibody (MdoD_33-351_ (MdoD_Δsignal_), C-terminally Myc-tagged). Polyclonal GroEL antibody was used to detect GroEL as a cytoplasmic control.

### Copurification assays to assess substrate binding to Tat core complexes

Purified full-length His_6_-SUMO-CueO_myc_ and His_6_-SUMO-MdoD_myc_ (purified as described above) were incubated with *S. cerevisiae* Ulp1 at 4 °C for 12 h and injected onto a Superose 6 Increase 10/300 GL size exclusion column (cytiva) pre-equilibrated in buffer C (20 mM HEPES pH 7.4, 150 mM NaCl, 0.02% GDN) to separate His-tagged SUMO from CueO or MdoD. Fractions corresponding to CueO or MdoD were collected and stored on ice and used the same day.

For experiments with *E. coli* TatBC complexes, membranes containing overexpressed His-tagged *E. coli* TatBC wild-type or its variants were prepared and solubilized in GDN as described in the ‘Purification of *E. coli* TatBC and TatABC complexes’ section. Insoluble material was removed by centrifugation (100,000*g*, 30 min), and clarified solubilized membranes were incubated with 200 μl of prewashed cOmplete His tag purification resin (Roche, 50% w/v slurry). The resin was washed with 15 CV of buffer (50 mM sodium phosphate pH 8.0, 150 mM NaCl, 2 mM imidazole, 1 mM EDTA, 0.02% (w/v) GDN). Purified CueO was then incubated with the resin-bound *E. coli* TatBC for 2 h. The resin was washed with 30 CV of buffer (50 mM sodium phosphate pH 8.0, 150 mM NaCl, 2 mM imidazole, 1 mM EDTA, 0.02% (w/v) GDN). Proteins were eluted from the resin with buffer (50 mM sodium phosphate pH 8.0, 150 mM NaCl, 200 mM imidazole, 1 mM EDTA, 0.02% (w/v) GDN). Proteins were separated by SDS–polyacrylamide gel electrophoresis (PAGE) and detected by immunoblotting using polyclonal antibodies against TatB, TatC and CueO.

For substrate-binding experiments with *N. salsuginis* Tat complexes *N. salsuginis* TatABC, TatBC and TatAC complexes were generated as follows. *N. salsuginis* TatABC was overexpressed in *E. coli* strain BL21 DE3 carrying both a pRSFDuet-1 vector containing *tatC*-strep (mcs1) and *tatB*-flag (mcs2) and a pACT7 vector containing *tatA*-(His)_6_. *N. salsuginis* TatBC was overexpressed in the *E. coli* strain BL21 DE3 carrying the pRSFDuet-1 vector containing *tatC*-strep (mcs1) and *tatB*-flag (mcs2). *N. salsuginis* TatAC was overexpressed in the *E. coli* strain BL21 DE3 carrying both a pRSFDuet-1 vector containing only *tatC*-strep (mcs1) and a pACT7 vector containing *tatA*-(His)_6_. Cells were grown in TB media containing kanamycin (50 μg ml^−1^) and chloramphenicol (20 μg ml^−1^) at 37 °C with shaking to A_600nm_ = 4.0, after which, cultures were supplemented with 0.1 mM IPTG and incubated for an additional 12 h at 25 °C with shaking. Cells were harvested by centrifugation (5,000*g*, 10 min). Membranes were prepared and solubilized in GDN as described in the ‘Purification of *E. coli* TatBC and TatABC complexes’ section. Insoluble material was removed by centrifugation (100,000*g*, 30 min) and clarified solubilised membranes were incubated with 200 μl of prewashed Streptactin XT superflow resin (50% w/v slurry, IBA Lifesciences). The resin was washed with 15 CV of buffer (50 mM sodium phosphate pH 8.0, 150 mM NaCl, 1 mM EDTA, 0.02% (w/v) GDN). Purified CueO or MdoD were incubated with resin-bound *N. salsuginis* TatABC, TatBC or TatAC for 2 h. The resin was washed with 30 CV of buffer (50 mM sodium phosphate pH 8.0, 150 mM NaCl, 1 mM EDTA, 0.02% (w/v) GDN). Proteins were eluted from the resin with buffer (50 mM sodium phosphate pH 8.0, 150 mM NaCl, 50 mM D-biotin, 1 mM EDTA, 0.02% (w/v) GDN). Proteins were separated by SDS–PAGE and detected by immunoblotting using antibodies against His tag (TatA), FLAG tag (TatB), strep tag (TatC) or Myc tag (MdoD or CueO).

### Gel quantification to determine TatB:TatC ratios

Purified TatABC (*E. coli* TatA_26(His)_6__TatBC) and TatBC (*E. coli* TatBC_(His)_6_) were loaded onto 4–20% SDS–PAGE gels at the following dilutions: 1, 0.75, 0.5 and 0.25. Gels were run at 180 V for 30–40 min and stained with Coomassie brilliant blue. Gels were imaged with a ChemiDoc MP imaging system (Bio-Rad), and gel bands were quantified using ImageJ. Quantification was performed with samples from three independent biological repeats.

### CG MD simulations

Membrane thinning was investigated using coarse-grained (CG) simulations. *The E. coli* TatBC complex cryo-EM structure was used as an input. The input protein was aligned according to the plane of the membrane with MEMEMBED^86^ and converted to a CG representation using the Martini 3 force field^87^. An elastic network with a force constant of 500 kJ mol^−1 ^nm^−2^ was used to restrain the secondary structure of the protein. A POPE:POPG bilayer at 1:4 molar ratio was built around the protein using the insane protocol^88^. The system was solvated with Martini 3 waters, and NaCl was added in a concentration of 150 mM to neutralize the system. The system was energy-minimized using the steepest descents method, followed by 10 ns equilibration in the NVT ensemble, then by 10 ns in the NPT ensemble, before 3× 5 μs production simulations. All production simulations sampled isothermic-isobaric ensembles at 310 K using the V-rescale thermostat (${\tau }_{T}$ = 1.0)^89^, the C-rescale barostat for semi-isotropic pressure coupling at 1.0 bar (${\tau }_{P}$ = 12.0)^90^ and a time-step of 20 fs. The reaction-field method was used to model long range electrostatic interactions. Bond lengths were constrained to their equilibrium values.

### Atomistic simulations

The cryo-EM structures of the TatBC complex, in the presence and absence of the MdoD signal peptide, were used as starting points for atomistic simulations. TatAC complexes were modelled by substituting TatA for TatB in the TatBC structures. Similarly, the TatBC–MdoD structure served as the basis for modelling the TatAC–MdoD complex. Atomistic simulations for all four system were prepared using the MemProtMD^91^ pipeline. After 1 μs, the CG systems were converted back to atomistic details using CG2AT^92^. The systems were further equilibrated for 1 ns maintaining the structure of the protein restrained. Three repeats of unrestrained 500-ns MD simulations were performed for each system. All simulations were performed in the isothermal–isobaric ensemble at 310 K and 1 bar using a time-step of 2 fs and the CHARMM36 force field^93^. Pressure was maintained at 1 bar using the C-rescale barostat (${\tau }_{P}$ = 1.0). Temperature was controlled using the velocity rescale thermostat (${\tau }_{T}$ = 0.1), with the solvent, lipids and protein coupled to an external bath. Electrostatics was described using the particle-mesh Ewald method^94^, with a cut-off of 1.2 nm, and the Van der Waals interactions were shifted between 1 and 1.2 nm. All MD simulations were performed using GROMACS 2023.4^95,96^.

### Statistics and reproducibility

All pull-down experiments shown are representative examples from *n* = 3 repeats (Figs. 3k, 4d,g and 5d,i). SDS resistance experiments are representative examples from *n* = 3 repeats (Figs. 3j and 5h). The preparation shown in gels Extended Data Fig. 10d,e was repeated once and the sample imaged and shown in Fig. 5e,f.

### Reporting summary

Further information on research design is available in the Nature Portfolio Reporting Summary linked to this article.

## Supplementary information

Supplementary Information Supplementary Figs. 1–6 and References.

Reporting summary

Supplementary Tables 1–5.

## Source data

Source Data Fig. 3 Unprocessed western blots and SDS resistance assays.

Source Data Fig. 4 Unprocessed western blots.

Source Data Fig. 5d–h Unprocessed western blots and SDS resistance assays.

Source Data Extended Data Fig. 10 Unprocessed western blots.

## Data Availability

Cryo-EM reconstructions and atomic models for *E. coli* TatBC (EMD-47343; PDB 9DZZ), *N. salsuginis* TatBC (EMD-47346; PDB 9E02), *M. xanthus* TatBC (EMD-47347; PDB 9E03), *E. coli* TatBC–MdoD (EMD-47345; PDB 9E01), *E. coli* MdoD (EMD-47352; PDB 9E08), *N. salsuginis* TatBC–CueO (EMD-47348; PDB 9E04), *N. salsuginis* TatBC in nanodisc (EMD-47350; PDB 9E06) and *N. salsuginis* TatAC (EMD-4735; PDB 9E07) have been deposited in the Electron Microscopy Data Bank and Protein Data Bank, respectively, with the appropriate accession codes listed in parenthesis beside each entry. The atomic model of *M. xanthus* TatC periplasmic domain solved by X-ray crystallography was deposited in the Protein Data Bank with accession code 9E0S. Source data are provided with this paper.

## References

[1] Barkan, A., Miles, D. & Taylor, W. C. Chloroplast gene expression in nuclear, photosynthetic mutants of maize. *EMBO J.* **5**, 1421–1427 (1986). 3743547 10.1002/j.1460-2075.1986.tb04378.x PMC1166961

[2] Palmer, T. & Berks, B. C. The twin-arginine translocation (Tat) protein export pathway. *Nat. Rev. Microbiol.* **10**, 483–496 (2012). 22683878 10.1038/nrmicro2814

[3] Berks, B. C., Palmer, T. & Sargent, F. The Tat protein translocation pathway and its role in microbial physiology. *Adv. Microb. Physiol.* **47**, 187–254 (2003). 14560665 10.1016/s0065-2911(03)47004-5

[4] Schafer, K. et al. The plant mitochondrial TAT pathway is essential for complex III biogenesis. *Current. Biol.* **30**, 840 (2020). 10.1016/j.cub.2020.01.001 32084398

[5] Petru, M. et al. Evolution of mitochondrial TAT translocases illustrates the loss of bacterial protein transport machines in mitochondria. *BMC Biol.* **16**, 141 (2018). 30466434 10.1186/s12915-018-0607-3 PMC6251230

[6] Celedon, J. M. & Cline, K. Intra-plastid protein trafficking: how plant cells adapted prokaryotic mechanisms to the eukaryotic condition. *Biochim. Biophys. Acta* **1833**, 341–351 (2013). 22750312 10.1016/j.bbamcr.2012.06.028 PMC3481018

[7] Moreira, D., Blaz, J., Kim, E. & Eme, L. A gene-rich mitochondrion with a unique ancestral protein transport system. *Curr. Biol.* **34**, 3812–3819 (2024). 39084221 10.1016/j.cub.2024.07.017

[8] Roger, A. J., Munoz-Gomez, S. A. & Kamikawa, R. The origin and diversification of mitochondria. *Curr. Biol.* **27**, R1177–R1192 (2017). 29112874 10.1016/j.cub.2017.09.015

[9] Rapoport, T. A., Li, L. & Park, E. Structural and mechanistic insights into protein translocation. *Annu. Rev. Cell Dev. Biol.* **33**, 369–390 (2017). 28564553 10.1146/annurev-cellbio-100616-060439

[10] Palmer, T. & Berks, B. C. Moving folded proteins across the bacterial cell membrane. *Microbiology* **149**, 547–556 (2003). 12634324 10.1099/mic.0.25900-0

[11] Cline, K. Mechanistic aspects of folded protein transport by the Tat system. *J. Biol. Chem.*10.1074/jbc.R114.626820 (2015). 10.1074/jbc.R114.626820 PMC4505407 25975269

[12] Hamsanathan, S. & Musser, S. M. The Tat protein transport system: intriguing questions and conundrums. *FEMS Microbiol. Lett.*10.1093/femsle/fny123 (2018). 10.1093/femsle/fny123 PMC5995166 29897510

[13] De Buck, E., Lammertyn, E. & Anne, J. The importance of the twin-arginine translocation pathway for bacterial virulence. *Trends Microbiol.* **16**, 442–453 (2008). 18715784 10.1016/j.tim.2008.06.004

[14] Berks, B. C. A common export pathway for proteins binding complex redox cofactors? *Mol. Microbiol.* **22**, 393–404 (1996). 8939424 10.1046/j.1365-2958.1996.00114.x

[15] Chaddock, A. M. et al. A new type of signal peptide: central role of a twin-arginine motif in transfer signals for the delta pH-dependent thylakoidal protein translocase. *EMBO J.* **14**, 2715–2722 (1995). 7796800 10.1002/j.1460-2075.1995.tb07272.x PMC398390

[16] Stanley, N. R., Palmer, T. & Berks, B. C. The twin arginine consensus motif of Tat signal peptides is involved in Sec-independent protein targeting in *Escherichia coli*. *J. Biol. Chem.* **275**, 11591–11596 (2000). 10766774 10.1074/jbc.275.16.11591

[17] Alcock, F. & Berks, B. C. New insights into the Tat protein transport cycle from characterizing the assembled Tat translocon. *Mol. Microbiol.* **118**, 637–651 (2022). 36151601 10.1111/mmi.14984 PMC10092561

[18] Blummel, A. S. et al. Structural features of the TatC membrane protein that determine docking and insertion of a twin-arginine signal peptide. *J. Biol. Chem.* **292**, 21320–21329 (2017). 29089385 10.1074/jbc.M117.812560 PMC5766949

[19] Rollauer, S. E. et al. Structure of the TatC core of the twin-arginine protein transport system. *Nature* **492**, 210–214 (2012). 23201679 10.1038/nature11683 PMC3573685

[20] Tarry, M. J. et al. Structural analysis of substrate binding by the TatBC component of the twin-arginine protein transport system. *Proc. Natl Acad. Sci. USA* **106**, 13284–13289 (2009). 19666509 10.1073/pnas.0901566106 PMC2718047

[21] Zoufaly, S. et al. Mapping precursor-binding site on TatC subunit of twin arginine-specific protein translocase by site-specific photo cross-linking. *J. Biol. Chem.* **287**, 13430–13441 (2012). 22362773 10.1074/jbc.M112.343798 PMC3339946

[22] Holzapfel, E. et al. The entire N-terminal half of TatC is involved in twin-arginine precursor binding. *Biochemistry* **46**, 2892–2898 (2007). 17300178 10.1021/bi062205b

[23] Ma, X. & Cline, K. Mapping the signal peptide binding and oligomer contact sites of the core subunit of the pea twin arginine protein translocase. *Plant Cell* **25**, 999–1015 (2013). 23512851 10.1105/tpc.112.107409 PMC3634702

[24] Alami, M. et al. Differential interactions between a twin-arginine signal peptide and its translocase in *Escherichia coli*. *Mol. Cell* **12**, 937–946 (2003). 14580344 10.1016/s1097-2765(03)00398-8

[25] Berks, B. C., Sargent, F. & Palmer, T. The Tat protein export pathway. *Mol. Microbiol.* **35**, 260–274 (2000). 10652088 10.1046/j.1365-2958.2000.01719.x

[26] Sargent, F. et al. Overlapping functions of components of a bacterial Sec-independent protein export pathway. *EMBO J.* **17**, 3640–3650 (1998). 9649434 10.1093/emboj/17.13.3640 PMC1170700

[27] Bogsch, E. G. et al. An essential component of a novel bacterial protein export system with homologues in plastids and mitochondria. *J. Biol. Chem.* **273**, 18003–18006 (1998). 9660752 10.1074/jbc.273.29.18003

[28] Settles, A. M. et al. Sec-independent protein translocation by the maize Hcf106 protein. *Science* **278**, 1467–1470 (1997). 9367960 10.1126/science.278.5342.1467

[29] Rodriguez, F. et al. Structural model for the protein-translocating element of the twin-arginine transport system. *Proc. Natl Acad. Sci. USA* **110**, E1092–E1101 (2013). 23471988 10.1073/pnas.1219486110 PMC3607022

[30] Zhang, Y., Wang, L., Hu, Y. & Jin, C. Solution structure of the TatB component of the twin-arginine translocation system. *Biochim. Biophys. Acta* **1838**, 1881–1888 (2014). 24699374 10.1016/j.bbamem.2014.03.015

[31] Hu, Y., Zhao, E., Li, H., Xia, B. & Jin, C. Solution NMR structure of the TatA component of the twin-arginine protein transport system from gram-positive bacterium *Bacillus subtilis*. *J. Am. Chem. Soc.* **132**, 15942–15944 (2010). 20726548 10.1021/ja1053785

[32] Ramasamy, S., Abrol, R., Suloway, C. J. & Clemons, W. M. Jr. The glove-like structure of the conserved membrane protein TatC provides insight into signal sequence recognition in twin-arginine translocation. *Structure* **21**, 777–788 (2013). 23583035 10.1016/j.str.2013.03.004 PMC3653977

[33] Cline, K. & Mori, H. Thylakoid DeltapH-dependent precursor proteins bind to a cpTatC-Hcf106 complex before Tha4-dependent transport. *J. Cell Biol.* **154**, 719–729 (2001). 11502764 10.1083/jcb.200105149 PMC2196467

[34] Alcock, F. et al. Assembling the Tat protein translocase. *Elife*10.7554/eLife.20718 (2016). 10.7554/eLife.20718 PMC5201420 27914200

[35] Bolhuis, A., Mathers, J. E., Thomas, J. D., Barrett, C. M. & Robinson, C. TatB and TatC form a functional and structural unit of the twin-arginine translocase from *Escherichia coli*. *J. Biol. Chem.* **276**, 20213–20219 (2001). 11279240 10.1074/jbc.M100682200

[36] Habersetzer, J. et al. Substrate-triggered position switching of TatA and TatB during Tat transport in *Escherichia coli*. *Open Biol.*10.1098/rsob.170091 (2017). 10.1098/rsob.170091 PMC5577447 28814647

[37] Severi, E., Bunoro Batista, M., Lannoy, A., Stansfeld, P. J. & Palmer, T. Characterization of a TatA/TatB binding site on the TatC component of the *Escherichia coli* twin arginine translocase. *Microbiology*10.1099/mic.0.001298 (2023). 10.1099/mic.0.001298 PMC10197872 36790402

[38] Alcock, F. et al. Live cell imaging shows reversible assembly of the TatA component of the twin-arginine protein transport system. *Proc. Natl Acad. Sci. USA* **110**, E3650–E3659 (2013). 24003141 10.1073/pnas.1306738110 PMC3780885

[39] Dabney-Smith, C., Mori, H. & Cline, K. Oligomers of Tha4 organize at the thylakoid Tat translocase during protein transport. *J. Biol. Chem.* **281**, 5476–5483 (2006). 16407186 10.1074/jbc.M512453200

[40] Mori, H. & Cline, K. A twin arginine signal peptide and the pH gradient trigger reversible assembly of the thylakoid ΔpH/Tat translocase. *J. Cell Biol.* **157**, 205–210 (2002). 11956224 10.1083/jcb.200202048 PMC2199252

[41] Cline, K. & McCaffery, M. Evidence for a dynamic and transient pathway through the TAT protein transport machinery. *EMBO J.* **26**, 3039–3049 (2007). 17568769 10.1038/sj.emboj.7601759 PMC1914107

[42] Bruser, T. & Sanders, C. An alternative model of the twin arginine translocation system. *Microbiol. Res.* **158**, 7–17 (2003). 12608575 10.1078/0944-5013-00176

[43] McNeilage, R., Ganesan, I., Keilman, J. & Theg, S. M. Cell-penetrating peptides stimulate protein transport on the Twin-arginine translocation pathway. Preprint at *b**ioRxiv*10.1101/2023.07.08.548235 (2024).

[44] Asher, A. H. & Theg, S. M. Electrochromic shift supports the membrane destabilization model of Tat-mediated transport and shows ion leakage during Sec transport. *Proc. Natl Acad. Sci. USA*10.1073/pnas.2018122118 (2021). 10.1073/pnas.2018122118 PMC8000419 33723047

[45] Celedon, J. M. & Cline, K. Stoichiometry for binding and transport by the twin arginine translocation system. *J. Cell Biol.* **197**, 523–534 (2012). 22564412 10.1083/jcb.201201096 PMC3352945

[46] Sharma, A., Chowdhury, R. & Musser, S. M. Oligomerization state of the functional bacterial twin-arginine translocation (Tat) receptor complex. *Commun. Biol.* **5**, 988 (2022). 36123532 10.1038/s42003-022-03952-2 PMC9485244

[47] Cleon, F. et al. The TatC component of the twin-arginine protein translocase functions as an obligate oligomer. *Mol. Microbiol.*10.1111/mmi.13106 (2015). 10.1111/mmi.13106 PMC5102672 26112072

[48] De Geyter, J. et al. Protein folding in the cell envelope of *Escherichia coli*. *Nat. Microbiol.* **1**, 16107 (2016). 27573113 10.1038/nmicrobiol.2016.107

[49] Wojnowska, M., Gault, J., Yong, S. C., Robinson, C. V. & Berks, B. C. Precursor–receptor interactions in the twin arginine protein transport pathway probed with a new receptor complex preparation. *Biochemistry* **57**, 1663–1671 (2018). 29460615 10.1021/acs.biochem.8b00026 PMC5852461

[50] Strauch, E. M. & Georgiou, G. *Escherichia coli* tatC mutations that suppress defective twin-arginine transporter signal peptides. *J. Mol. Biol.* **374**, 283–291 (2007). 17936785 10.1016/j.jmb.2007.09.050 PMC2661573

[51] Kneuper, H. et al. Molecular dissection of TatC defines critical regions essential for protein transport and a TatB-TatC contact site. *Mol. Microbiol.* **85**, 945–961 (2012). 22742417 10.1111/j.1365-2958.2012.08151.x PMC3712464

[52] Buchanan, G. et al. Functional complexity of the twin-arginine translocase TatC component revealed by site-directed mutagenesis. *Mol. Microbiol.* **43**, 1457–1470 (2002). 11952898 10.1046/j.1365-2958.2002.02853.x

[53] Motouchi, S., Kobayashi, K., Nakai, H. & Nakajima, M. Identification of enzymatic functions of osmo-regulated periplasmic glucan biosynthesis proteins from *Escherichia coli* reveals a novel glycoside hydrolase family. *Commun. Biol.* **6**, 961 (2023). 37735577 10.1038/s42003-023-05336-6 PMC10514313

[54] James, M. J., Coulthurst, S. J., Palmer, T. & Sargent, F. Signal peptide etiquette during assembly of a complex respiratory enzyme. *Mol. Microbiol.* **90**, 400–414 (2013). 23961722 10.1111/mmi.12373

[55] Rodrigue, A., Chanal, A., Beck, K., Muller, M. & Wu, L. F. Co-translocation of a periplasmic enzyme complex by a hitchhiker mechanism through the bacterial tat pathway. *J. Biol. Chem.* **274**, 13223–13228 (1999). 10224080 10.1074/jbc.274.19.13223

[56] Jack, R. L., Sargent, F., Berks, B. C., Sawers, G. & Palmer, T. Constitutive expression of *Escherichia coli* tat genes indicates an important role for the twin-arginine translocase during aerobic and anaerobic growth. *J. Bacteriol.* **183**, 1801–1804 (2001). 11160116 10.1128/JB.183.5.1801-1804.2001 PMC95070

[57] Sargent, F. et al. Purified components of the *Escherichia coli* Tat protein transport system form a double-layered ring structure. *Eur. J. Biochem.* **268**, 3361–3367 (2001). 11422364 10.1046/j.1432-1327.2001.02263.x

[58] Ize, B., Stanley, N. R., Buchanan, G. & Palmer, T. Role of the *Escherichia coli* Tat pathway in outer membrane integrity. *Mol. Microbiol.* **48**, 1183–1193 (2003). 12787348 10.1046/j.1365-2958.2003.03504.x

[59] Gerard, F. & Cline, K. The thylakoid proton gradient promotes an advanced stage of signal peptide binding deep within the Tat pathway receptor complex. *J. Biol. Chem.* **282**, 5263–5272 (2007). 17172598 10.1074/jbc.M610337200

[60] Gerard, F. & Cline, K. Efficient twin arginine translocation (Tat) pathway transport of a precursor protein covalently anchored to its initial cpTatC binding site. *J. Biol. Chem.* **281**, 6130–6135 (2006). 16407185 10.1074/jbc.M512733200

[61] Li, L. et al. Crystal structure of a substrate-engaged SecY protein-translocation channel. *Nature* **531**, 395–399 (2016). 26950603 10.1038/nature17163 PMC4855518

[62] Aldridge, C., Ma, X., Gerard, F. & Cline, K. Substrate-gated docking of pore subunit Tha4 in the TatC cavity initiates Tat translocase assembly. *J. Cell Biol.* **205**, 51–65 (2014). 24711501 10.1083/jcb.201311057 PMC3987133

[63] Frobel, J., Blummel, A. S., Drepper, F., Warscheid, B. & Muller, M. Surface-exposed domains of TatB involved in the structural and functional assembly of the Tat translocase in *Escherichia coli*. *J. Biol. Chem.* **294**, 13902–13914 (2019). 31341014 10.1074/jbc.RA119.009298 PMC6755809

[64] Blummel, A. S., Haag, L. A., Eimer, E., Muller, M. & Frobel, J. Initial assembly steps of a translocase for folded proteins. *Nat. Commun.* **6**, 7234 (2015). 26068441 10.1038/ncomms8234 PMC4490388

[65] Frobel, J., Rose, P. & Muller, M. Twin-arginine-dependent translocation of folded proteins. *Philos. Trans. R. Soc. Lond. B* **367**, 1029–1046 (2012). 22411976 10.1098/rstb.2011.0202 PMC3297433

[66] New, C. P., Ma, Q. & Dabney-Smith, C. Routing of thylakoid lumen proteins by the chloroplast twin arginine transport pathway. *Photosynth. Res.* **138**, 289–301 (2018). 30101370 10.1007/s11120-018-0567-z

[67] Hao, B., Zhou, W. & Theg, S. M. Hydrophobic mismatch is a key factor in protein transport across lipid bilayer membranes via the Tat pathway. *J. Biol. Chem.* **298**, 101991 (2022). 35490783 10.1016/j.jbc.2022.101991 PMC9207671

[68] Hou, B., Heidrich, E. S., Mehner-Breitfeld, D. & Bruser, T. The TatA component of the twin-arginine translocation system locally weakens the cytoplasmic membrane of *Escherichia coli* upon protein substrate binding. *J. Biol. Chem.* **293**, 7592–7605 (2018). 29535185 10.1074/jbc.RA118.002205 PMC5961041

[69] Stockwald, E. R. et al. Length matters: functional flip of the short TatA transmembrane helix. *Biophys. J.* **122**, 2125–2146 (2023). 36523158 10.1016/j.bpj.2022.12.016 PMC10257086

[70] Mehner-Breitfeld, D. et al. TatA and TatB generate a hydrophobic mismatch important for the function and assembly of the Tat translocon in *Escherichia coli*. *J. Biol. Chem.* **298**, 102236 (2022). 35809643 10.1016/j.jbc.2022.102236 PMC9424591

[71] Wu, X. & Rapoport, T. A. Translocation of proteins through a distorted lipid bilayer. *Trends Cell Biol.* **31**, 473–484 (2021). 33531207 10.1016/j.tcb.2021.01.002 PMC8122044

[72] Hoffmann, S., Schmidt, C., Walter, S., Bender, J. K. & Gerlach, R. G. Scarless deletion of up to seven methyl-accepting chemotaxis genes with an optimized method highlights key function of CheM in *Salmonella* Typhimurium. *PLoS ONE* **12**, e0172630 (2017). 28212413 10.1371/journal.pone.0172630 PMC5315404

[73] Battye, T. G., Kontogiannis, L., Johnson, O., Powell, H. R. & Leslie, A. G. iMOSFLM: a new graphical interface for diffraction-image processing with MOSFLM. *Acta Crystallogr. D* **67**, 271–281 (2011). 21460445 10.1107/S0907444910048675 PMC3069742

[74] Terwilliger, T. C. et al. Decision-making in structure solution using Bayesian estimates of map quality: the PHENIX AutoSol wizard. *Acta Crystallogr. D* **65**, 582–601 (2009). 19465773 10.1107/S0907444909012098 PMC2685735

[75] Terwilliger, T. C. Automated main-chain model building by template matching and iterative fragment extension. *Acta Crystallogr. D* **59**, 38–44 (2003). 12499537 10.1107/S0907444902018036 PMC2745878

[76] Brown, A. et al. Tools for macromolecular model building and refinement into electron cryo-microscopy reconstructions. *Acta Crystallogr. D* **71**, 136–153 (2015). 25615868 10.1107/S1399004714021683 PMC4304694

[77] Afonine, P. V. et al. Real-space refinement in PHENIX for cryo-EM and crystallography. *Acta Crystallogr. D* **74**, 531–544 (2018). 10.1107/S2059798318006551 PMC6096492 29872004

[78] Hagn, F., Nasr, M. L. & Wagner, G. Assembly of phospholipid nanodiscs of controlled size for structural studies of membrane proteins by NMR. *Nat. Protoc.* **13**, 79–98 (2018). 29215632 10.1038/nprot.2017.094 PMC5806515

[79] Caesar, J. et al. SIMPLE 3.0. Stream single-particle cryo-EM analysis in real time. *J. Struct. Biol. X* **4**, 100040 (2020). 33294840 10.1016/j.yjsbx.2020.100040 PMC7695977

[80] Punjani, A., Zhang, H. & Fleet, D. J. Non-uniform refinement: adaptive regularization improves single-particle cryo-EM reconstruction. *Nat. Methods* **17**, 1214–1221 (2020). 33257830 10.1038/s41592-020-00990-8

[81] Zivanov, J., Nakane, T. & Scheres, S. H. W. A Bayesian approach to beam-induced motion correction in cryo-EM single-particle analysis. *IUCrJ* **6**, 5–17 (2019). 30713699 10.1107/S205225251801463X PMC6327179

[82] Asarnow, D., Palovcak, E. & Cheng, Y. asarnow/pyem: UCSF pyem v0.5 (v0.5). *Zenodo*10.5281/zenodo.3576630 (2019).

[83] Williams, C. J. et al. MolProbity: more and better reference data for improved all-atom structure validation. *Protein Sci.* **27**, 293–315 (2018). 29067766 10.1002/pro.3330 PMC5734394

[84] Kelley, L. A., Mezulis, S., Yates, C. M., Wass, M. N. & Sternberg, M. J. The Phyre2 web portal for protein modeling, prediction and analysis. *Nat. Protoc.* **10**, 845–858 (2015). 25950237 10.1038/nprot.2015.053 PMC5298202

[85] Pettersen, E. F. et al. UCSF ChimeraX: structure visualization for researchers, educators, and developers. *Protein Sci.* **30**, 70–82 (2021). 32881101 10.1002/pro.3943 PMC7737788

[86] Nugent, T. & Jones, D. T. Membrane protein orientation and refinement using a knowledge-based statistical potential. *BMC Bioinformatics* **14**, 276 (2013). 24047460 10.1186/1471-2105-14-276 PMC3852961

[87] Souza, P. C. T. et al. Martini 3: a general purpose force field for coarse-grained molecular dynamics. *Nat. Methods* **18**, 382–388 (2021). 33782607 10.1038/s41592-021-01098-3 PMC12554258

[88] Wassenaar, T. A., Ingolfsson, H. I., Bockmann, R. A., Tieleman, D. P. & Marrink, S. J. Computational lipidomics with insane: a versatile tool for generating custom membranes for molecular simulations. *J. Chem. Theory Comput.* **11**, 2144–2155 (2015). 26574417 10.1021/acs.jctc.5b00209

[89] Bussi, G., Donadio, D. & Parrinello, M. Canonical sampling through velocity rescaling. *J. Chem. Phys.* **126**, 014101 (2007). 17212484 10.1063/1.2408420

[90] Bernetti, M. & Bussi, G. Pressure control using stochastic cell rescaling. *J. Chem. Phys.* **153**, 114107 (2020). 32962386 10.1063/5.0020514

[91] Stansfeld, P. J. et al. MemProtMD: automated insertion of membrane protein structures into explicit lipid membranes. *Structure* **23**, 1350–1361 (2015). 26073602 10.1016/j.str.2015.05.006 PMC4509712

[92] Vickery, O. N. & Stansfeld, P. J. CG2AT2: an enhanced fragment-based approach for serial multi-scale molecular dynamics simulations. *J. Chem. Theory Comput.* **17**, 6472–6482 (2021). 34492188 10.1021/acs.jctc.1c00295 PMC8515810

[93] Huang, J. & MacKerell, A. D. Jr. CHARMM36 all-atom additive protein force field: validation based on comparison to NMR data. *J. Comput. Chem.* **34**, 2135–2145 (2013). 23832629 10.1002/jcc.23354 PMC3800559

[94] Darden, T., York, D. & Pedersen, L. Particle mesh Ewald—an *N*.log(*N*) method for Ewald sums in large systems. *J. Chem. Phys.* **98**, 10089–10092 (1993).

[95] Abraham, M. J. et al. GROMACS: high performance molecular simulations through multi-level parallelism from laptops to supercomputers. *SoftwareX* **1-2**, 19–25 (2015).

[96] GROMACS 2023.4 source code (GROMACS, 2023).

