# Supplementary material for: Structure and substrate recognition by the bacterial twin-arginine translocation (Tat) core complex
Source: Nat Microbiol. 2026 Jun 22;11(7):2047–64. doi: 10.1038/s41564-026-02399-z (PMC13323110; doi:10.1038/s41564-026-02399-z)
Supplement: Supplementary file 1 — Supplementary Figs. 1–6 and References. [file 41564_2026_2399_MOESM1_ESM.pdf]

nature microbiology

Supplementary information https://doi.org/10.1038/s41564-026-02399-z

Structure and substrate recognition by the bacterial twin-arginine translocation (Tat) core complex

In the format provided by the authors and unedited

Supplementary Information For:

Title:

Structure and substrate recognition by the bacterial Twin-arginine translocation (Tat) core complex

Authors: Justin C. Deme[1,2,3,]†, Owain J. Bryant[1,4,5]†, Mariana Batista[6], Phill Stansfeld[6], Ben C. Berks[4,]*, Susan M. Lea[1,2,3,5]*

Affiliations:

[1]Center for Structural Biology, Center for Cancer Research, National Cancer Institute, Frederick, MD 21702, USA.

[2]Sir William Dunn School of Pathology, University of Oxford, Oxford, UK.

[3]Central Oxford Structural Molecular Imaging Centre, University of Oxford, Oxford, UK.

[4]Department of Biochemistry, University of Oxford, Oxford, OX1 3QU, UK.

[5]Structural Biology, St Jude Children's Research Hospital, Memphis, TN 38105, USA.

[6]School of Life Sciences, University of Warwick, Coventry, CV47 7AL, UK.

*Correspondence to: susan.lea@stjude.org or ben.berks@bioch.ox.ac.uk

†These authors contributed equally.

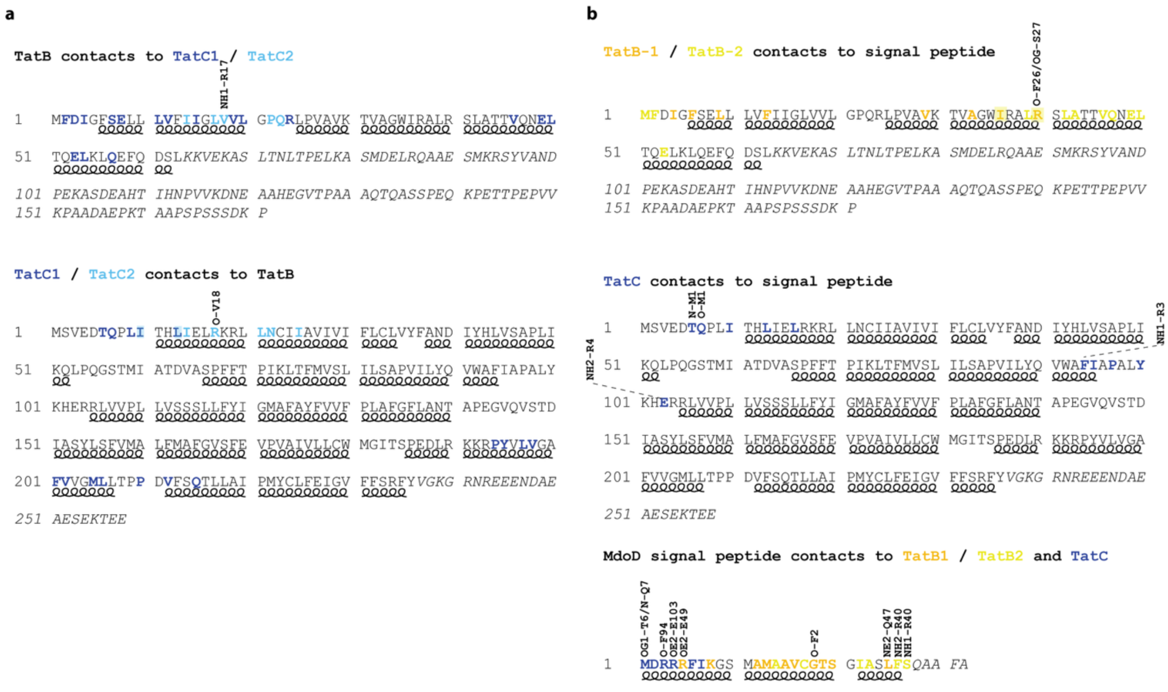

c

```
TatB  1    MFDIGFSELL LVFIIGLVVL GPQRLPVAVK TVAGWIRALR SLATTVQNEL
TatA  1    MGGISIWQLL IIAVIVVLLF GTKKLGSIGS DLGASIKGFK KAMSDDEPKQ


TatB 51   TQELKLQEFQ DSL
TatA 51   DKTSQDADFT AKT
```

Supplementary Figure 1. Mapping *E. coli* core complex interactions onto the protein sequences.

Residues involved in helices are denoted by a helix cartoon beneath the sequence. Residues not seen in the structures are in italics. Colour indicates which chain is involved in the contact. **a**, Contacts between *E. coli* TatB and TatC. Coloured residues indicate which copy of TatC is contacted. Atoms in the other chain to which the coloured residue forms a hydrogen-bond are shown vertically. **b**, Contacts to the MdoD signal peptide. Coloured residues denote which copy of TatB is involved and the signal peptide residues are coloured to denote the Tat chain contacted. **c**, TatA and TatB are aligned from the N-terminus with the ordered portion of TatB shown. Residues coloured in TatB are involved in contacts with signal peptide. Of these 15 TatB residues only 4 are identical in TatA (indicated in bold).

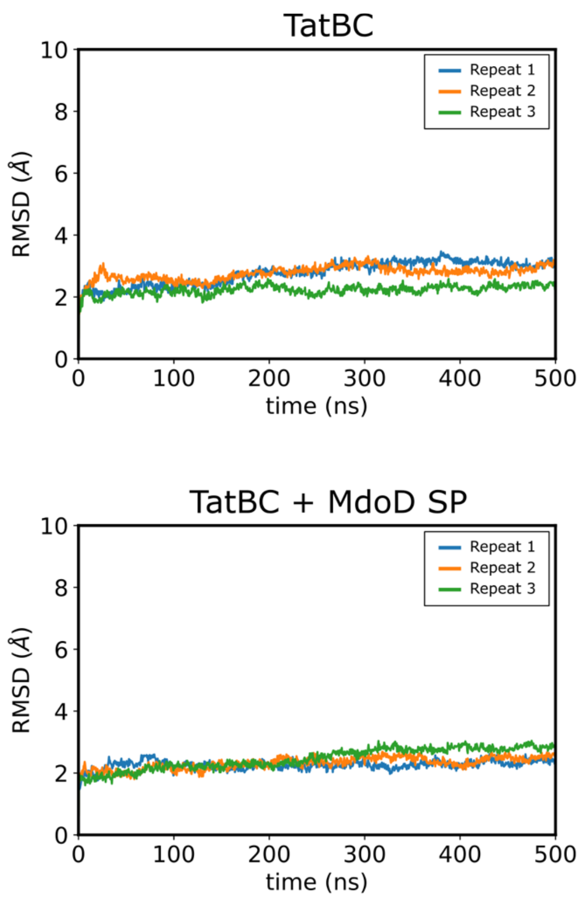

Supplementary Figure 2. Repeated simulations reveal all structures are stable on a 500ns time scale.

Full atomistic simulations of each structure embedded in a lipid bilayer were repeated 3 times and demonstrate that all systems remain stable throughout the simulations.

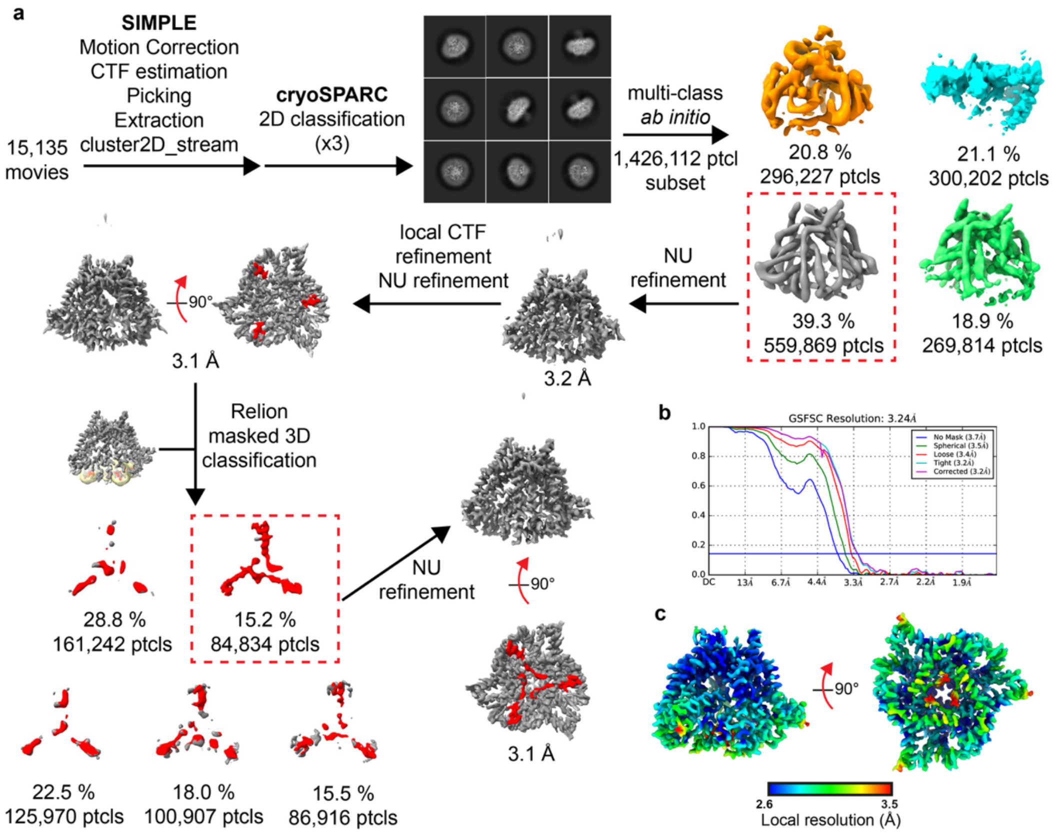

Supplementary Figure 3. Cryo-EM workflow for *N. salsuginis* TatBC-CueO signal peptide complex.

a, Image processing workflow. **b**, Gold-standard Fourier Shell Correlation (FSC) curves used for global resolution estimation. **c**, Local resolution estimation of the volume

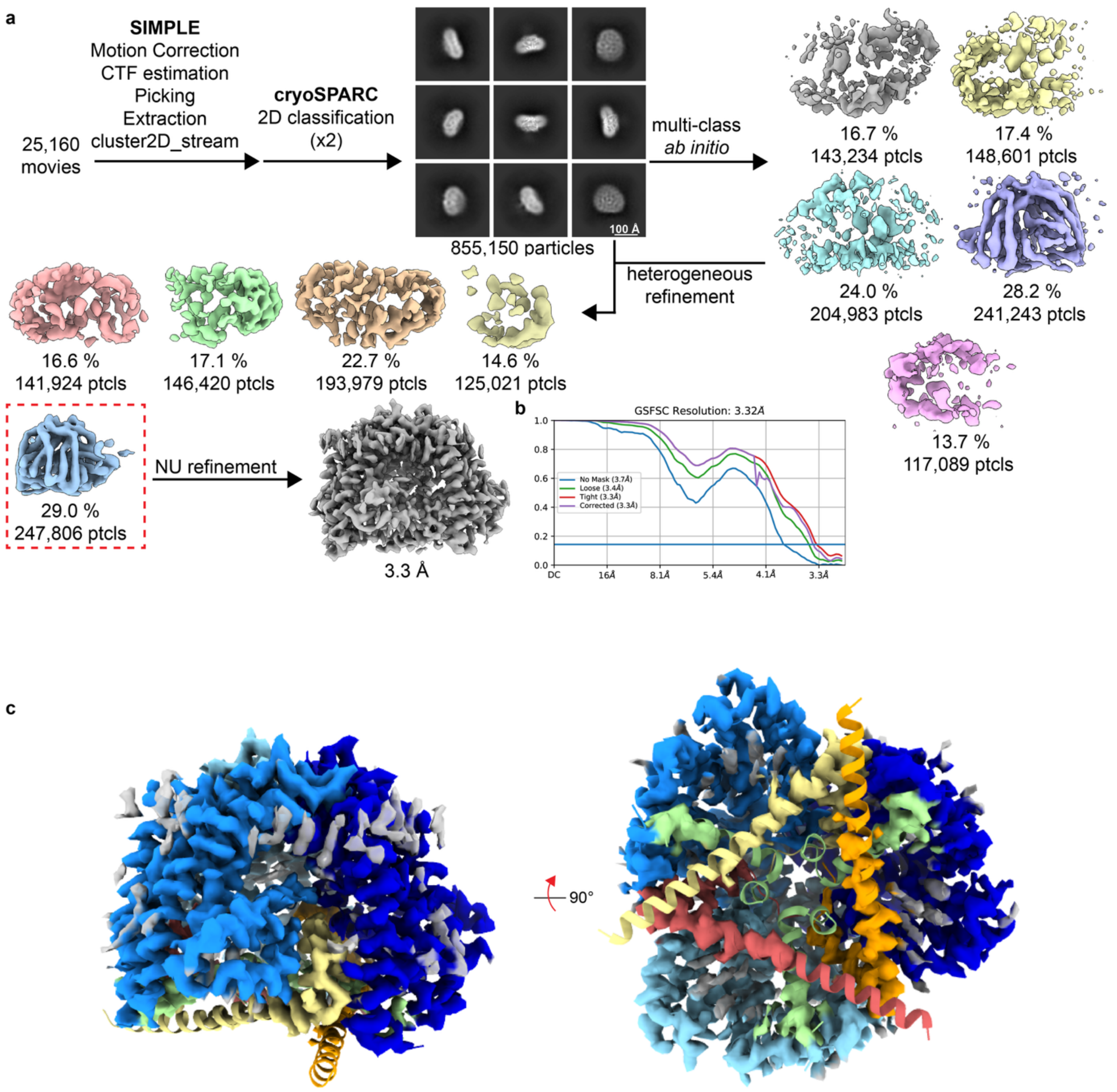

Supplementary Figure 4. Asymmetric C1 reconstruction of an *E. coli* TatABC-substrate complex.

An unsymmetrized C1 reconstruction. The TatABC complex was purified first by pulling on a twin-strep tag on TatC then via an internal his-tag at position 26 of TatA to which CueO substrate was added prior to grid preparation. **a**, Image processing workflow. **b**, Gold-standard Fourier Shell Correlation (FSC) curves used for global resolution estimation. **c**, The C1 volume is not appreciably asymmetric at the resolution of the complex (3.3 Å). The volume is essentially indistinguishable from the TatBC-MdoD complex (coordinates shown as docked cartoon representation) except that the amphipathic helices of TatB and the substrate peptide are substantially less well ordered in the TatABC-CueO volume implying lower occupancy and/or higher mobility of these regions.

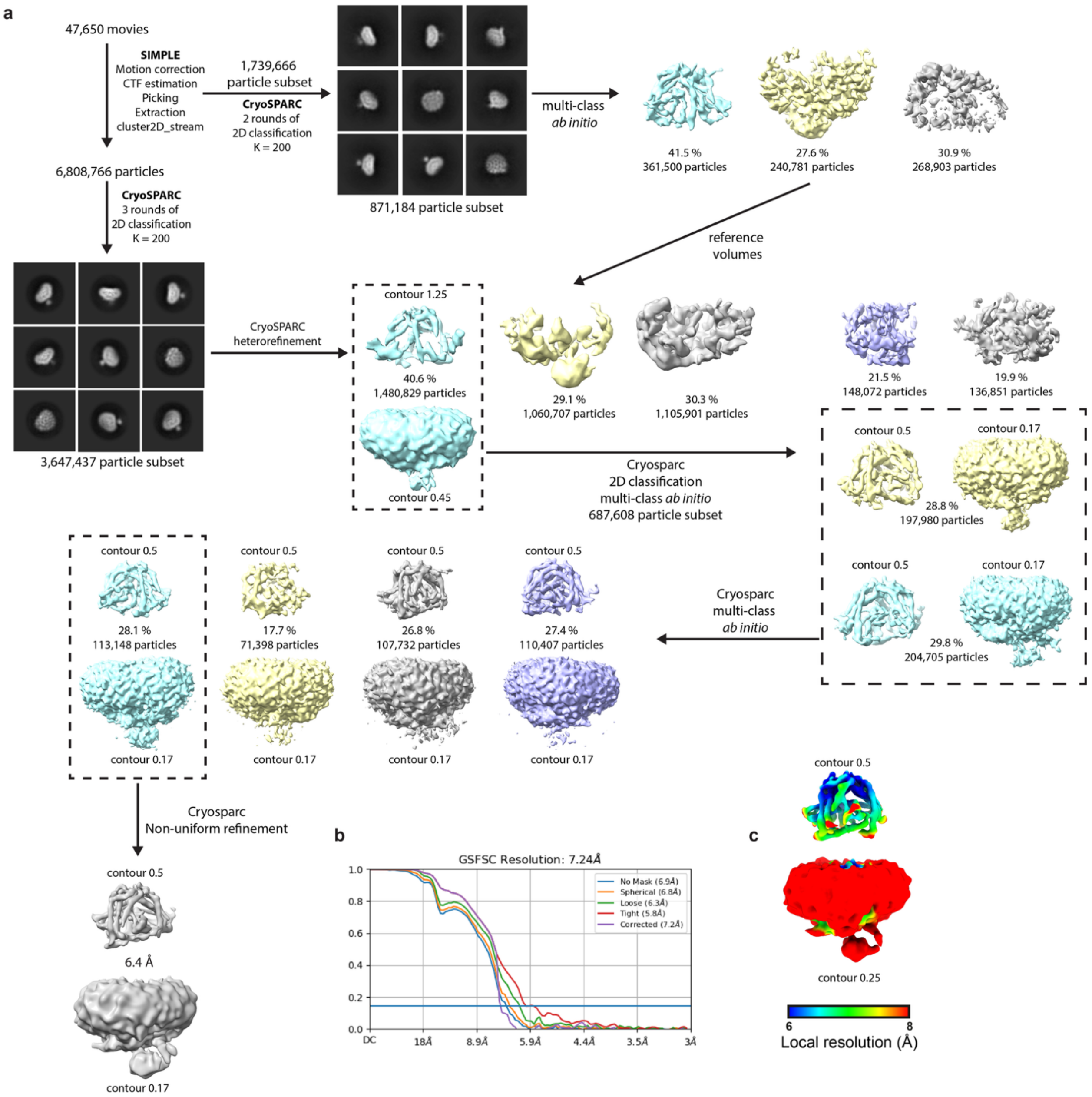

Supplementary Figure 5. Cryo-EM workflow for an *E. coli* TatA_ALFA_BC-nanobody complex.

a, Image processing workflow. **b**, Gold-standard Fourier Shell Correlation (FSC) curves used for global resolution estimation. **c**, Local resolution estimation of the volume

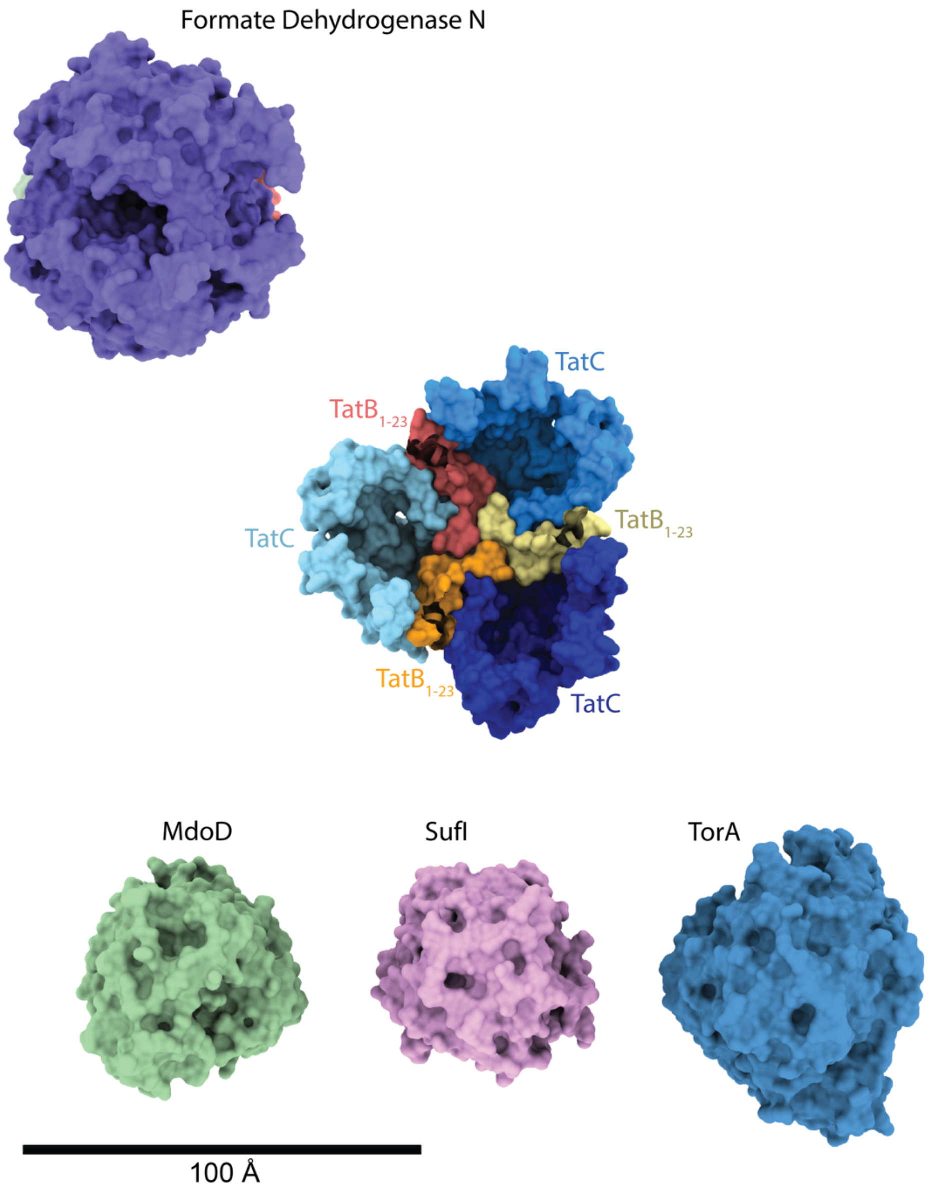

Supplementary Figure 6. Comparison of the sizes of the folded domains of a selection of Tat substrates relative to the size of the TatBC complex.

A surface representation of TatBC viewed from the cytoplasm is shown in the centre of the figure (coloured as in main text figures) with the amphipathic helices of TatB removed. Surface representations of four substrates are shown below - with each substrate oriented to present the minimal cross-section for transport. Substrates are shown as full assemblies e.g. MdoD is a homodimer and formate dehydrogenase N is a FDNGH heterodimer) None of these substrates could be accommodated within the TatBC complex without structural rearrangement that would essentially equate to complete disassembly of the TatBC complex.

Supplementary References

1 Baumgarten, T. *et al*. Isolation and characterization of the E. coli membrane protein production strain Mutant56(DE3). *Sci Rep* **7**, 45089 (2017). https://doi.org/10.1038/srep45089

2 Studier, F. W. & Moffatt, B. A. Use of bacteriophage T7 RNA polymerase to direct selective high-level expression of cloned genes. *J Mol Biol* **189**, 113-130 (1986). https://doi.org/10.1016/0022-2836(86)90385-2

3 Casadaban, M. J. & Cohen, S. N. Lactose genes fused to exogenous promoters in one step using a Mu-lac bacteriophage: in vivo probe for transcriptional control sequences. *Proc Natl Acad Sci U S A* **76**, 4530-4533 (1979).

4 Wexler, M. *et al*. TatD is a cytoplasmic protein with DNase activity. No requirement for TatD family proteins in sec-independent protein export. *J Biol Chem* **275**, 16717-16722 (2000). https://doi.org/10.1074/jbc.M000800200

5 Leahy, D. J., Hendrickson, W. A., Aukhil, I. & Erickson, H. P. Structure of a fibronectin type III domain from tenascin phased by MAD analysis of the selenomethionyl protein. *Science* **258**, 987-991 (1992). https://doi.org/10.1126/science.1279805

6 Tarry, M. J. *et al*. Structural analysis of substrate binding by the TatBC component of the twin-arginine protein transport system. *Proc Natl Acad Sci U S A* **106**, 13284-13289 (2009). https://doi.org/10.1073/pnas.0901566106

7 McDevitt, C. A., Hicks, M. G., Palmer, T. & Berks, B. C. Characterisation of Tat protein transport complexes carrying inactivating mutations. *Biochem Biophys Res Commun* **329**, 693-698 (2005). https://doi.org/10.1016/j.bbrc.2005.02.038

8 Zamenhof, P. J. & Villarejo, M. Construction and properties of Escherichia coli strains exhibiting -complementation of -galactosidase fragments in vivo. *J Bacteriol* **110**, 171-178 (1972). https://doi.org/10.1128/jb.110.1.171-178.1972

9 Guzman, L. M., Belin, D., Carson, M. J. & Beckwith, J. Tight regulation, modulation, and high-level expression by vectors containing the arabinose PBAD promoter. *J Bacteriol* **177**, 4121-4130 (1995). https://doi.org/10.1128/jb.177.14.4121-4130.1995

10 Hoffmann, S., Schmidt, C., Walter, S., Bender, J. K. & Gerlach, R. G. Scarless deletion of up to seven methyl-accepting chemotaxis genes with an optimized method highlights key function of CheM in Salmonella Typhimurium. *PLoS One* **12**, e0172630 (2017). https://doi.org/10.1371/journal.pone.0172630

11 Drew, D., Lerch, M., Kunji, E., Slotboom, D. J. & de Gier, J. W. Optimization of membrane protein overexpression and purification using GFP fusions. *Nat Methods* **3**, 303-313 (2006). https://doi.org/10.1038/nmeth0406-303

12 Alcock, F. *et al*. Assembling the Tat protein translocase. *Elife* **5** (2016). https://doi.org/10.7554/eLife.20718
