# Supplementary figures and images for: Structure and substrate recognition by the bacterial twin-arginine translocation (Tat) core complex
Source: Nat Microbiol. 2026 Jun 22;11(7):2047–64. doi: 10.1038/s41564-026-02399-z (PMC13323110; doi:10.1038/s41564-026-02399-z)

Main\_Figure\_3j

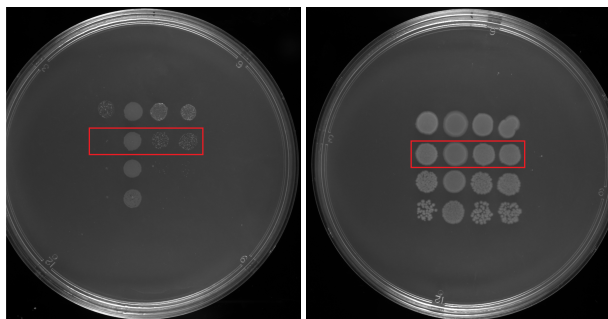

Main\_Figure\_3k

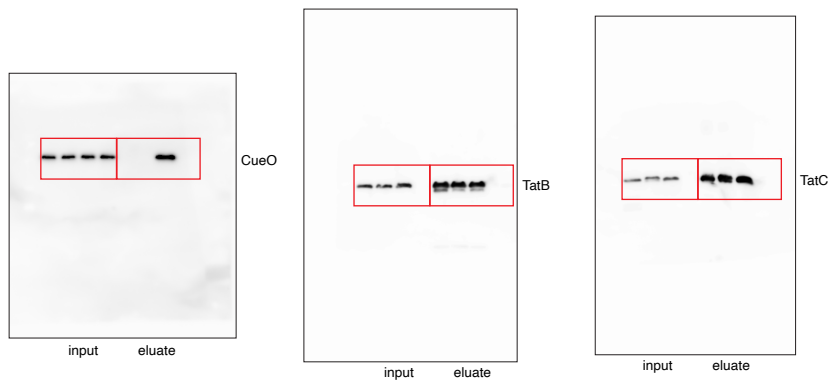

Supplement: Supplementary file 4 — Unprocessed western blots and SDS resistance assays. [file 41564_2026_2399_MOESM4_ESM.pdf]

Main\_Figure\_4d

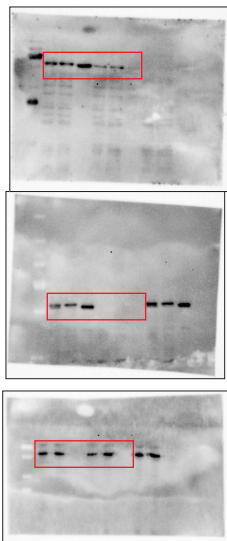

Main\_Figure\_4g

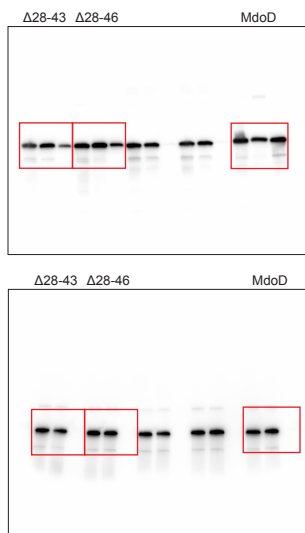

Supplement: Supplementary file 5 — Unprocessed western blots. [file 41564_2026_2399_MOESM5_ESM.pdf]

**Main\_Figure\_5d**

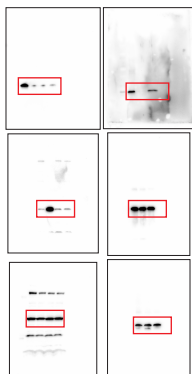

**Main\_Figure\_5h**

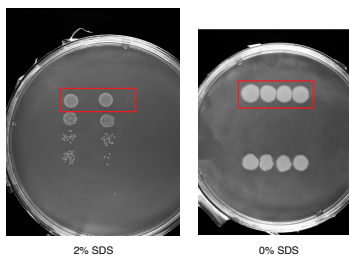

**Main\_Figure\_5i**

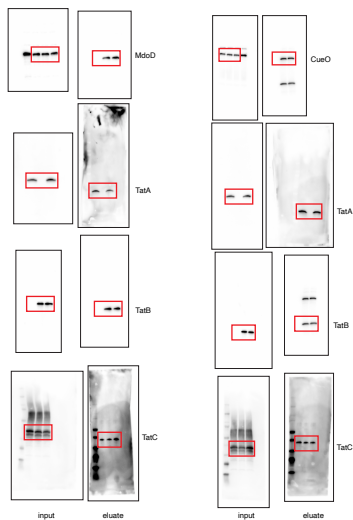

Supplement: Supplementary file 6 — Unprocessed western blots and SDS resistance assays. [file 41564_2026_2399_MOESM6_ESM.pdf]

Extended\_Figure\_10d

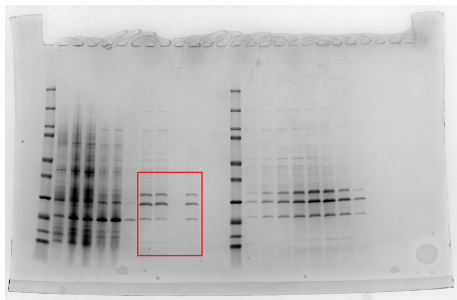

Extended\_Figure\_10e

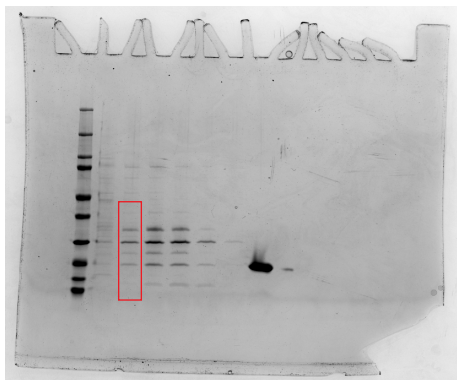

Supplement: Supplementary file 7 — Unprocessed western blots. [file 41564_2026_2399_MOESM7_ESM.pdf]
